# High-Voltage Stabilization of O3-Type Layered Oxide for Sodium-Ion Batteries by Simultaneous Tin Dual Modification

**DOI:** 10.1021/acs.chemmater.2c00522

**Published:** 2022-04-29

**Authors:** Tengfei Song, Lin Chen, Dominika Gastol, Bo Dong, José F. Marco, Frank Berry, Peter Slater, Daniel Reed, Emma Kendrick

**Affiliations:** † School of Metallurgy and Materials, University of Birmingham, Edgbaston, Birmingham B15 2TT, U.K.; ‡ School of Chemistry, University of Birmingham, Edgbaston, Birmingham B15 2TT, U.K.; § Instituto de Química Física ″Rocasolano″, CSIC, Serrano 119, Madrid 28006, Spain; ∥ The Faraday Institution, Harwell Science and Innovation Campus, Didcot OX11 0RA, U.K.

## Abstract

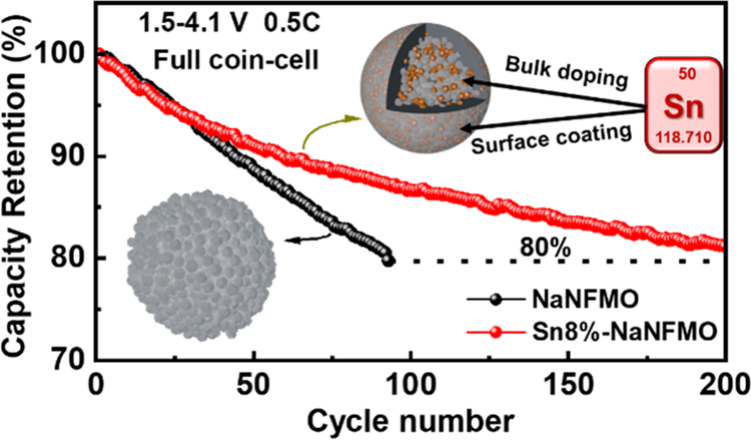

O3-type layered oxide materials are considered to be a highly suitable cathode for sodium-ion batteries (NIBs) due to their appreciable specific capacity and energy density. However, rapid capacity fading caused by serious structural changes and interfacial degradation hampers their use. A novel Sn-modified O3-type layered NaNi_1/3_Fe_1/3_Mn_1/3_O_2_ cathode is presented, with improved high-voltage stability through simultaneous bulk Sn doping and surface coating in a scalable one-step process. The bulk substitution of Sn^4+^ stabilizes the crystal structure by alleviating the irreversible phase transition and lattice structure degradation and increases the observed average voltage. In the meantime, the nanolayer Sn/Na/O composite on the surface effectively inhibits surface parasitic reactions and improves the interfacial stability during cycling. A series of Sn-modified materials are reported. An 8%-Sn-modified NaNi_1/3_Fe_1/3_Mn_1/3_O_2_ cathode exhibits a doubling in capacity retention increase after 150 cycles in the wide voltage range of 2.0–4.1 V *vs* Na/Na^+^ compared to none, and 81% capacity retention is observed after 200 cycles in a full cell *vs* hard carbon. This work offers a facile process to simultaneously stabilize the bulk structure and interface for the O3-type layered cathodes for sodium-ion batteries and raises the possibility of similar effective strategies to be employed for other energy storage materials.

## 1 Introduction

With the widespread increase in the numbers of batteries needed for consumer electronics, electric vehicles, and energy storage industries, lithium-ion batteries are experiencing an unprecedented period of rapid development. However, the relatively low reserves and uneven distribution of widely used strategic metals and critical materials in LIBs such as lithium, nickel, cobalt, and graphite have forced researchers to search for alternatives.^1−3^ Sodium-ion batteries offer one such solution, as sodium is geographically widespread and can be harvested through seawater and from rock salt and is significantly more abundant than lithium (Na: 23 000 ppm *vs* Li: 17 ppm in the Earth’s crust).^4,5^ In addition, alternative transition metals, such as Mn,^6,7^ Fe,^8,9^ Cu,^10,11^ Ti,^12^ Cr,^13^ V,^14,15^ can be utilized, instead of cobalt for the cathode, along with the reported safety benefits.^16−18^ Therefore, sodium-ion batteries (NIBs) offer a reasonable low-cost sustainable alternative to current lithium-ion batteries. However, the much higher atomic weight of Na (Na 23 g/mol *vs* Li 6.9 g/mol) and higher standard electrochemical potential make it difficult for NIBs to surpass LIBs in terms of energy density.^14,19^

Cathode materials are primarily responsible for the key performance indicators of batteries such as energy density, cycling performance, and cost; therefore, the development of new cathode materials is required for NIBs to achieve the required capacity and structural stability at a low cost.^20^ O3-type layered oxides (Na*_x_*TMO_2_, 0. 8<*x* ≤ 1; TM = Fe, Cr, Co, Mn, Ni, V, Cu, or mixtures of them) are considered as one of the most appealing candidates because of their high theoretical capacity, appropriate operating potential, and simple synthesis processes.^21−23^ However, these types of crystal structures often suffer from multistructural evolutions during the Na^+^ extraction/insertion process compared to their Li analogues, induced by the larger ionic radius of sodium ions (Na 1.02 Å *vs* Li 0.76 Å), especially at high voltage, resulting in deteriorating host crystal structures and fading in cycling stability.^24−26^

Among various reported Na*_x_*TMO_2_ materials, cobalt-free O3-type layered NaNi_1/3_Fe_1/3_Mn_1/3_O_2_ is one of the most promising cathode materials for NIBs, since first reported by the Johnson group.^9^ This material illustrates high reversibility and undergoes an O3-P3-O3 phase change during the desodiation/sodiation process in the voltage range of 2.0–4.0 V (*vs* Na/Na^+^); this *R*3̅*m* layered structure remains intact after more than 100 cycles.^9,27^ However, when the cut-off voltage is raised to higher than 4.0 V to pursue higher energy density, an irreversible phase change to O3′ and P3′ monoclinic phase occurs.^28^ This irreversible structural evolution mainly originates from the migration of TM ions and has been proven to be detrimental to stable cycling under higher charging potentials.^26,29^

As with many of these O3-type layered oxides, interfacial reactions between the cathode and electrolyte also accelerate the degradation of cathode material, causing capacity fading upon cycling.^30−32^ Surface degradation becomes more serious when operated at higher potentials.^33^ Given these aspects, methods to simultaneously suppress the phase transformation and also stabilize the particle surfaces at a higher cut-off voltage (>4.0 V) need to be investigated before they can be used in practice.^34^

Electrochemically inactive element substitution, such as Li,^35^ Cu,^36−38^ Mg,^39,40^ Ti,^41−44^ and Sn,^45−50^ has proven an effective strategy to modify the material’s bulk structure properties and improve the high-voltage structural stability of layered oxides. Among these elements, Sn is particularly promising because the binding energy of Sn–O (548 kJ mol^–1^) is much stronger than that of Ni–O (391.6 kJ mol^–1^), Fe–O (409 kJ mol^–1^), and Mn–O (402 kJ mol^–1^). Recent reports have shown that Sn substitution can smooth the charge/discharge voltage profile, by suppressing phase transitions and increasing the average working voltage.^47,51−53^ Therefore, the introduction of Sn^4+^ into NaNi_1/3_Fe_1/3_Mn_1/3_O_2_ may help to inhibit the TMO_2_ slab gliding and suppress the high-voltage irreversible phase transformation.

In this work, the incorporation of Sn into a NaNi_1/3_Fe_1/3_Mn_1/3_O_2_ cathode has been studied to simultaneously stabilize the crystal structure as well as the particle surface at high cell voltages. Through an extensive study of the structural changes by in-depth *in situ* and *ex situ* X-ray diffraction (XRD) characterization during Na^+^ intercalation/deintercalation, Sn is proven to be effective in stabilizing the layered structure by suppressing the irreversible phase transformation under the high-voltage region (∼4.1 V *vs* Na/Na^+^). In addition, the simultaneously formed nanocoating layer with sodium residues on the surface of the particles can improve the interfacial stability during cycling (as shown in Figure S1, ESI†). As a result, the structural stability and electrochemical performance of NaNi_1/3_Fe_1/3_Mn_1/3_O_2_ are significantly improved within the voltage range of 2.0–4.1 V *vs* Na/Na^+^. The modified cathode demonstrated excellent cycling stability as well as rate capability. The practical use of the modified electrode was further verified in a full cell with hard carbon as anode. The work illustrates that a combination of bulk doping and surface modification should be considered for the optimization of cathode performance and highlights that this combined beneficial effect can be achieved in a single scalable step.

## 2 Experimental Section

### 2.1 Materials Synthesis

Spherical [Ni_1/3_Fe_1/3_Mn_1/3_](OH)_2_ precursors were synthesized by a hydroxide coprecipitation method. First, stoichiometric amounts of NiSO_4_·6H_2_O (Alfa Aesar 98%), FeSO_4_·7H_2_O (Honeywell 99.9%), and MnSO_4_·H_2_O (VWR 99.5%) were uniformly mixed and dissolved into deionized water with a concentration of 2 M. This mixed metal solution was pumped into a continuously stirred tank reactor. At the same time, a 4 M NaOH (Alfa Aesar 98%) solution as a precipitant and 4 M NH_4_OH solution (28–30%, Sigma-Aldrich) as a chelating agent were also separately fed into the reactor in ambient air. The pump rates were set to achieve 1:1 mole ratio of NH_4_OH to transition metals. Stirring was done at 1000 rpm with a four-finned baffle. The temperature was 50 °C, and the pH was carefully controlled at 11.0 ± 0.1 by adjusting the NaOH pumping rate. After vigorous stirring for 12 h, the spherical [Ni_1/3_Fe_1/3_Mn_1/3_](OH)_2_ precursor was filtered and rinsed thoroughly with deionized water, dried in a vacuum oven at 100 °C overnight, and then sieved.

For the Sn-modified Na[Ni_1/3_Fe_1/3_Mn_1/3_]O_2_, to ensure low cost, fluorine and chlorine-free layered oxides, a tin oxide precursor was chosen rather than a soluble tin compound. This was added at the milling stage, so that the same hydroxide precursor could be utilized for all synthesis batches. The precursor, [Ni_1/3_Fe_1/3_Mn_1/3_](OH)_2_ was mixed with SnO_2_ nanoparticles (99.9%, Strem Chemicals) (1.0, 3.0, 5.0, 8.0, and 10.0 mol %) and then ball milled (Retsch, PM100CM) at 250 rpm for 4 h. Then, the SnO_2_-covered precursors were thoroughly mixed with the required Na_2_CO_3_ (99.9%, Sigma-Aldrich Co.) and ball milled at 250 rpm for 30 min. An excess of 5 mol % Na_2_CO_3_ was used to compensate for any volatilization of Na during calcination. The mixture was calcined to 550 °C for 6 h with a ramp rate of 5 °C min^–1^, followed by 900 °C for 9 h with a ramp rate of 2 °C min^–1^ in air, and then slowly cooled (1 °C min^–1^) to 150 °C. Finally, the layered Sn-modified materials (Figure S2, ESI†) were removed from the furnace and transferred into an argon-filled glovebox. For Sn-free pristine Na[Ni_1/3_Fe_1/3_Mn_1/3_]O_2_ material, the same synthetic process without the addition of SnO_2_ was conducted. The obtained samples with different contents of Sn are named as NaNFMO, Sn1%-NaNFMO, Sn3%-NaNFMO, Sn5%-NaNFMO, Sn8%-NaNFMO, and Sn10%-NaNFMO, respectively.

### 2.2 Analytical Techniques

The structural characteristics of the as-prepared materials before and after cycling were determined by X-ray diffraction using Bruker D8 Advance instrument with Cu Kα radiation source. The diffracted data were recorded at a scan rate of 1° min^–1^ in the 2θ range between 10 and 120° at 40 KV and 30 mA. *In situ* XRD was carried out in an E-cell *in situ* cell, equipped with a beryllium window. The morphology of the samples was observed by scanning electron microscopy coupled with energy-dispersive spectroscopy (EDS) (Philips XL-30 FEG ESEM). Cross-sectional SEM/EDS images were acquired by dual beam SEM FEI Quanta 3D FEG FIB-SEM. Selected area electron diffraction (SAED) patterns and high-resolution transmission electron microscopy (HRTEM) images were obtained with a JEOL JEM-F200 electron microscope operated at 200 kV. The precise chemical compositions of the prepared cathode materials were examined by inductively coupled plasma atomic emission spectrometry (ICP-OES, Agilent Technologies). Each sample was dissolved in 2 mL of aqua regia solution, which was then diluted using 2% HNO_3_ before measurements. Raman spectra were recorded on a Renishaw InVia Raman spectrometer with a laser wavelength of 488 nm. The surface composition and valence of TM ions from the particle surface to the inner were determined by X-ray photoelectron spectroscopy (XPS). All data were recorded under a vacuum of better than 1·10^–9^ mbar with a PHOIBOS 150 electron analyzer (SPECS) using Al Kα radiation (1486.6 eV) and a constant pass energy of 100 and 20 eV for the wide and narrow scans, respectively. The binding energy scale was referenced to the main C 1s contribution of the adventitious carbon layer, which was set at 284.6 eV. All of the spectra were computer-fitted using pseudo-Voight profiles (30% Lorentzian-70% Gaussian) and a Shirley background. The binding energies are accurate within ±0.2 eV. ^57^Fe Mössbauer spectroscopy data were recorded at room temperature using a conventional constant acceleration spectrometer and a ^57^Co (Rh) source. The velocity scale was calibrated using an α-Fe foil 6 μm thick. All of the spectra were computer-fitted, and the isomer shifts referred to the centroid of the spectrum of α-Fe at room temperature.

### 2.3 Electrochemical Performance Tests

The electrochemical characterizations were evaluated using CR2032 coin-type half-cells assembled in an argon-filled glovebox. The cathodes were prepared by mixing 87 wt % active material, 5.8 wt % carbon black (TimCal, C65), 0.2 wt % multiwalled carbon nanotubes (MWCNT), 1.0 wt % oxalic acid, and 6.0 wt % polyvinylidene fluoride (PVDF 5130, Solvay) in *N*-methyl-2-pyrrolidine (NMP) and then coated the mixture onto an aluminum foil. After drying at 120 °C for 24 h in vacuum, the electrode disks (14.8 mm) were punched and weighed. The mass loading of active materials on cathodes was 6–7 mg cm^–2^. Hard carbon (Kuranode, Kuraray) anodes were made for the full cell with the ratio of 90% active, 5% PVDF (5130, Solvay), and 5% conductive additive (C45, Timcal) in a similar manner to the cathode. For the Na metal anode, a sodium metal disk was cut from sodium cubes (Aldrich, 99%), and then it was rolled and punched out. The electrolyte consists of 1 M NaPF_6_ in EC/DEC (1/1, V/V) with 1 vol % FEC (fluoroethylene carbonate) as an electrolyte additive. Twenty-five μm thick polypropylene polymer (pp) (2325, Celgard) was used as the separator. Galvanostatic charge/discharge, cyclic voltammetry (CV), electrochemical impedance spectroscopy (EIS) measurements, and galvanostatic intermittent titration technique (GITT) measurement were performed on a BCS battery test system. For the GITT test, the cells were charged at 15 mA g^–1^ (0.1C) for 5 min and then relax for 1.5 h. The effective surface area S was assumed to be the surface area from active material particles in the electrode, as used previously.^54^ For the EIS measurements, all cells were tested in the frequency range from 1 MHz to 1 mHz with an amplitude of 5 mA after being charged to 4.1 V *vs* Na^+^/Na.

## 3 Results and Discussion

### 3.1 Materials Characterization

The detailed chemical compositions of the prepared samples were measured by ICP-OES, as shown in Table S1 (ESI†). The experimental Ni/Fe/Mn/Sn molar ratios are in good agreement with the expected stoichiometry within the error of the determination. As expected, ∼5% Na is lost during firing, as shown in the final elemental ratio.

**Figure 1:**
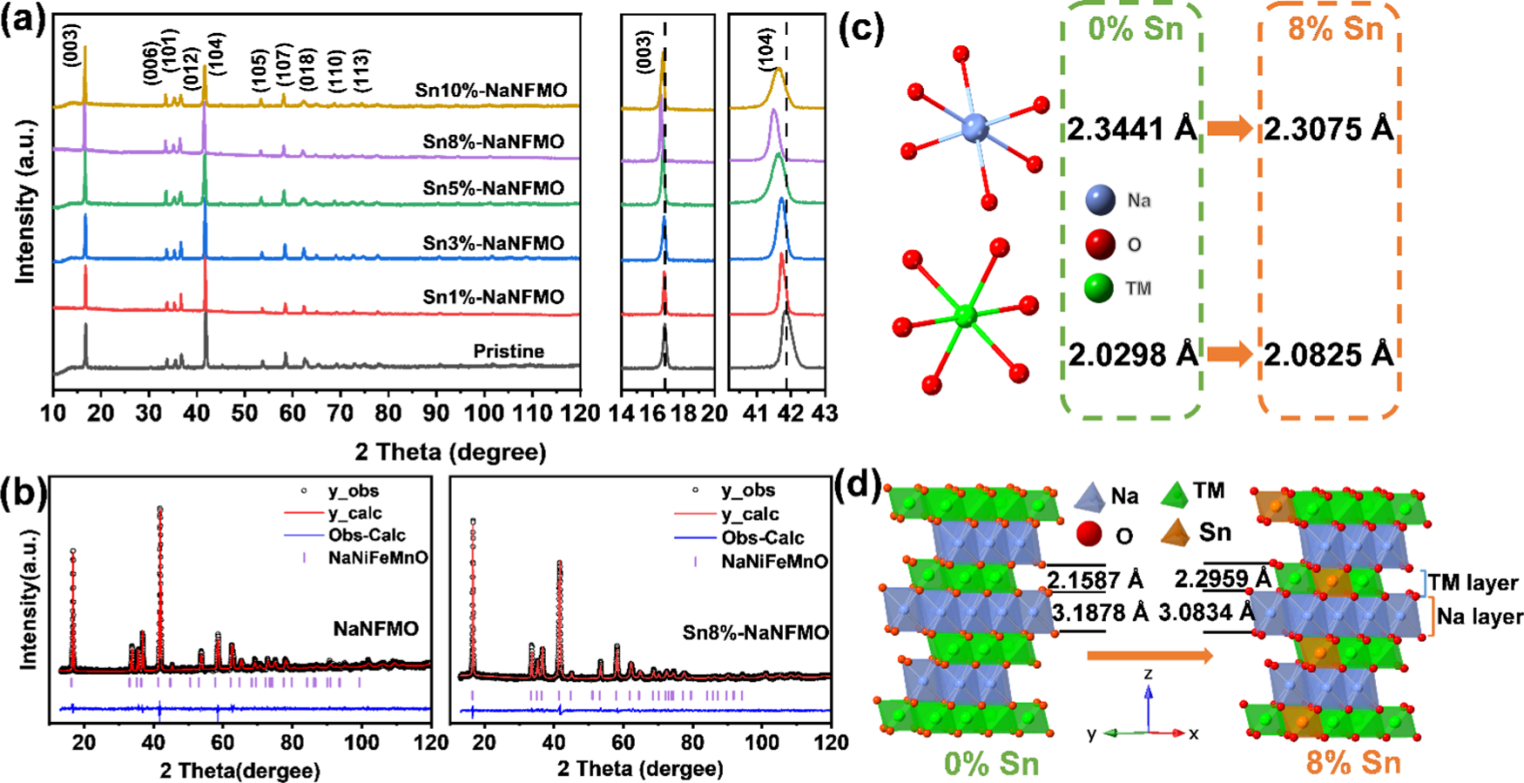
Structural characterization of the as-synthesized materials. (a) XRD patterns of all samples; Rietveld refinement results of (b) NaNFMO (left) and Sn8%-NaNFMO (right); and (c, d) Na–O–TM structure evolution before and after 8% Sn modification.

The crystal structures of the prepared samples have been characterized by XRD, and the results are presented in Figure 1a. A single-phase material is produced in all cases that can be indexed to a hexagonal O3-type α-NaFeO_2_ structure (R3̅ m space group). No impurity peaks of SnO_2_ are observed in the XRD patterns for the Sn-modified samples (Figure S3, ESI†). From the magnification of the (003) and (104) reflections, we can see that both peaks shift toward a lower Bragg angle with increasing Sn content, and Sn8%-NaNFMO presents the largest shift, indicating the largest expansion of the interplanar spacing as shown in Figure 1d (*c* axis).

Lattice parameters were calculated using the powder profile refinement program GSAS based on the initial structural model of O3 NaMO_2_ (Figures 1b, S4, and S5, ESI†), and the refined crystallographic parameters are summarized in Table S2 (ESI†). As shown in Figure S5, the lattice parameter *a* gradually increases with increasing Sn content, confirming that Sn^4+^ has entered into the crystal structure successfully. The lattice parameter *c* is first observed to decrease when the doping amount is 1%, due to the stronger binding energy of Sn–O than that of Ni–O, Fe–O, and Mn–O (as described in the Introduction section), which will strengthen the bonding between TM and O and thus shrinking the *d*-spacing. However, further increasing Sn-doping levels above 1% results in lattice parameter c increase, which could be attributed to the larger radius of Sn^4+^ (0.69Å) compared to Mn^4+^ (0.53 Å) and Fe^3+^ (0.645 Å).^43^

Modulating on O3 NaNFMO via Sn substitution also leads to the expansion of the TMO_2_ slab (from 2.1587 to 2.2959 Å) and contraction of Na layer spacing (from 3.1878 to 3.0834 Å), associated with the increase in average TM–O bond length, and enhancement of the binding energy between Na and O as illustrated in Figure 1c,d and Table S2 (ESI†). The change of localized electronic structure upon desodiation/sodiation is considered as the origin of Na^+^/vacancy ordering and the resulting phase transitions.^17,55−57^ The enlarged transition-metal layers may be beneficial for intensifying the degree of electronic delocalization and thus suppress monoclinic phase transition under the high-voltage region and improve electrochemical properties.^55^ This effect is confirmed through *in situ* XRD studies and GITT measurements discussed later.

Additionally, the intensity ratio of *I*_003_/*I*_104_ is a sensitive parameter for indicating the degree of cation mixing. With increasing TM–O bond ionicity, it is more difficult for TM ions to migrate into alternate layers; thus, the value of *I*_003_/*I*_104_ dramatically increases from 0.753 for 0% Sn to 1.657 for 10% Sn (Figure S5, ESI†), indicating that the degree of cation mixing decreases with increasing Sn content. However, it is worth noting that when the doping amount reaches 10%, the peaks become broadened and reverse back to a higher angle, as evidenced by the d-104 peak. The broadening of the peaks indicates the possibility of having multiple phases with close lattice parameters, which may be due to the sudden increase of Na vacancy (Table S3), leading to internal stress and lattice distortion.

The local structure evolution induced by Sn modification was further analyzed by Raman spectroscopy. As shown in Figure S6 (ESI†), both samples show three obvious bonds near 580, 490, and 340 cm^–1^, which originated from the A_1g_ and E_1g_ modes of TM–O vibrations and the E_2g_ mode of Na vibrations of the layered hexagonal *R*3̅*m* symmetry, respectively.^58−60^ No peak related to Sn–O was found.^61^ The intensity of the three bonds all increased after Sn substitution, which indicates that the fraction that contributed to the vibrations is increased, and the distortions of the crystal structure decreased. This result also indicates that Sn^4+^ ions had been successfully incorporated into the lattice.

The morphology and elemental distribution of Sn-modified samples were analyzed by SEM and HRTEM techniques. NaNFMO and Sn8%-NaNFMO are elective as the representative samples. As shown in Figure S7a,b (ESI†), both two samples have good spherical secondary particles with an average diameter of ∼6 μm, and both spherical secondary particles are comprised of a great number of small primary particles with a diameter in a range of 100–500 nm. However, we can also observe that primary particles of the Sn-modified sample are significantly smaller than those without Sn, and the particles are more densely packed. In addition, the surface of the NaNFMO sphere has many voids, while the Sn-NaNFMO sphere is much smoother. This difference in morphology suggests that Sn modification may alter the surface properties, particle growth kinetics, and ultimately the final morphology of NaNFMO. The decreased size and enhanced integration could be better for shortening Na^+^ diffusion distance and improving the mechanical strength. The EDS mapping of Sn8%-NaNFMO in Figure S7c (ESI†) shows that the Sn element is uniformly distributed on the surface of the secondary particle.

**Figure 2:**
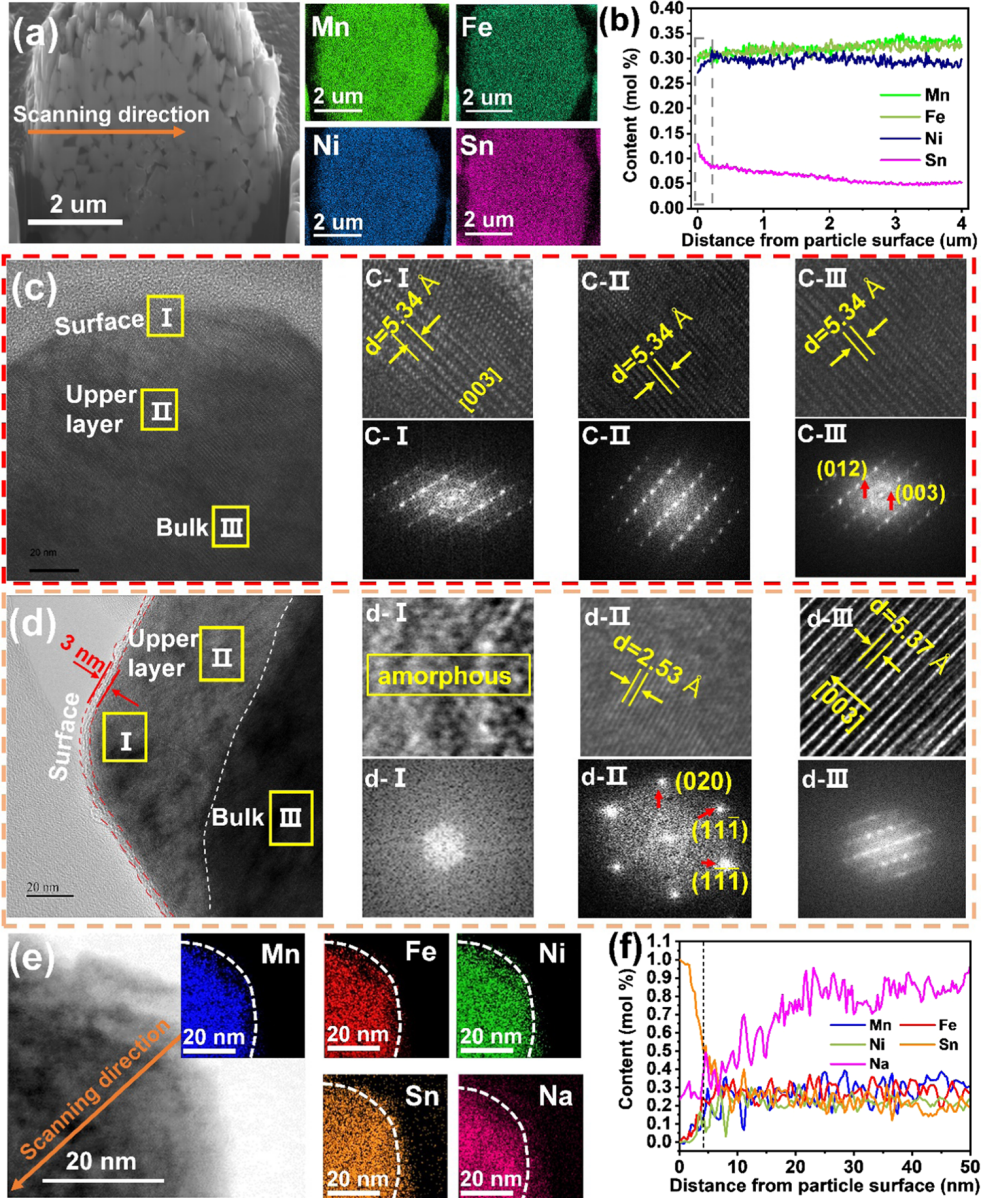
(a) Cross-sectional SEM image and EDS element mappings of the as-prepared Sn8%-NaNFMO acquired by FIB, and (b) corresponding counts content distribution of elements along the arrow; HRTEM and the corresponding FFT of (c) NaNFMO and (d) Sn8%-NaNFMO; and (e, f) STEM/EDS mapping and corresponding count content of element distribution in a single primary particle of the as-prepared Sn8%-NaNFMO.

To further evaluate the distribution of Sn, cross-sectional SEM and EDS line scans were used to detect any variation in the elemental distribution within the secondary particle. Ni, Fe, and Mn elements are relatively uniformly distributed within the particle, while Sn is observed in higher concentrations on the surface of the secondary particles compared to the core, as shown in Figure 2a,b. It is expected that the surface-enriched Sn is beneficial for improving-interfacial stability due to the stronger ionic bonding with oxygen.

Figure 2c,d shows the HRTEM images and corresponding fast Fourier transform (FFT) of NaNFMO and Sn8%-NaNFMO samples, respectively. NaNFMO sample demonstrates well-preserved layered lattice fringes in the selected regions with a crystal plane spacing of ∼5.34 Å, which can be assigned to a hexagonal phase with the R3̅m space group according to the FFT patterns and selected area electron diffraction (SAED) in Figure S8 (ESI†). By contrast, Sn8%-NaNFMO consists of three structures—from the outermost amorphous structure (≈3 nm) to the upper layer (10−100 nm ) with rock-salt structure, then to the inner layered structure. The complicated surface structure likely originates from the surface-enriched Sn, which forms a disordered arrangement of sodium and transition metal on the cation lattice.

**Figure 3:**
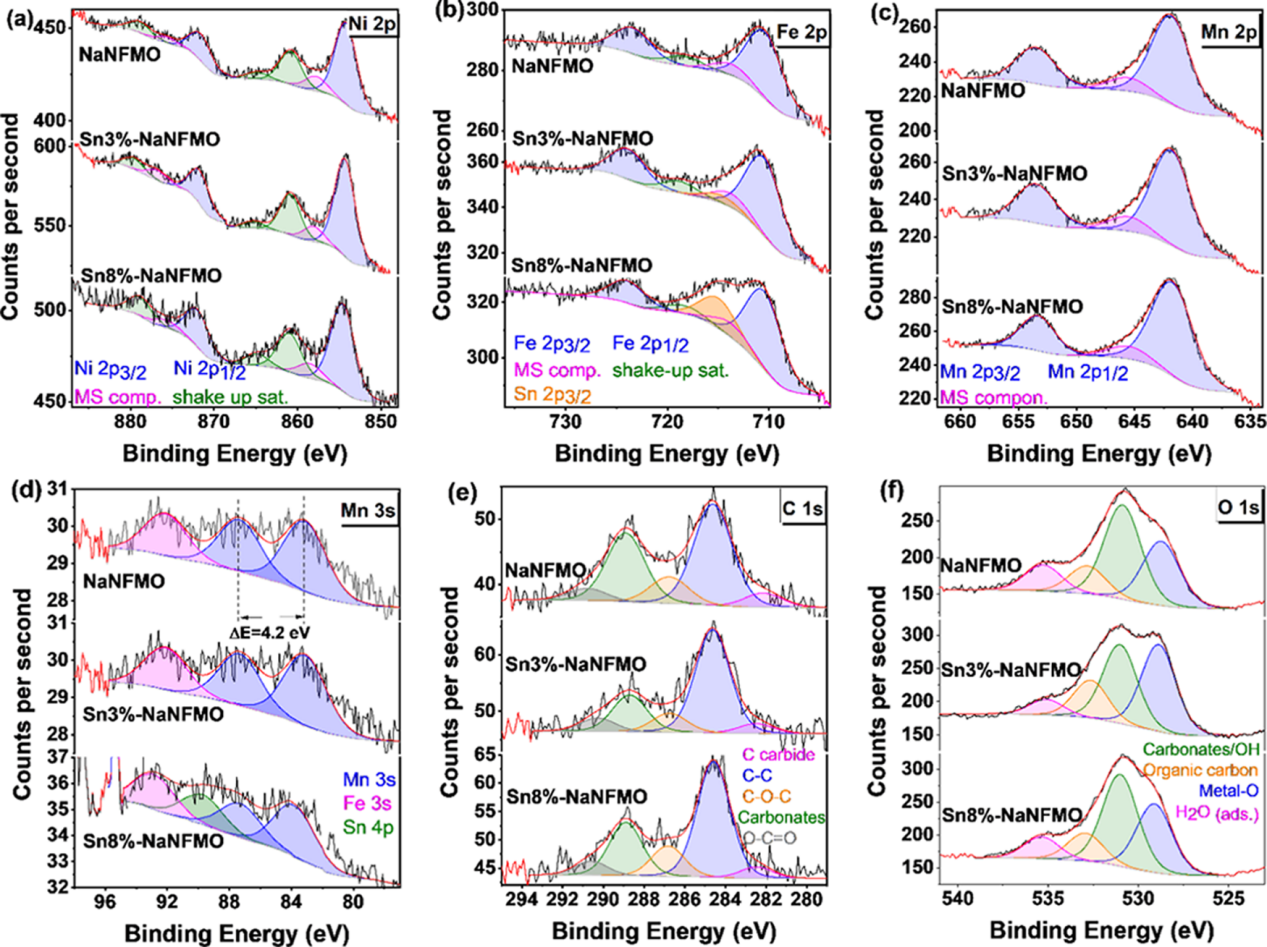
XPS spectra from the surface of NaNFMO particles with 0, 3, and 8% Sn additions: (a) Ni 2p, (b) Fe 2p, (c) Mn 2p, (d) Mn 3s, (e) C 1s, and (f) O 1s XPS spectra.

The surface composition of the particles is complex. Figure 2e,f shows the elemental mapping and the changes in elemental distribution from the surface of a single primary particle of the as-prepared Sn8%-NaNFMO. To show the distribution of the surface elements more clearly, a curve (white dotted line) is drawn with the range of the Mn element as a benchmark. Sn and Na elements are clearly observed on the outside layer with a thickness of several nanometers, giving direct evidence that the surface coating layer comprises both sodium and tin. XPS results in Figures 3b and S9a–c (discussed in detail below) show tin and sodium upon the surface also in these samples. Significant carbonate content is also observed upon the surface in all samples but more so in the nondoped sample. STEM indicates that the surface of the particles is amorphous in nature. Therefore, from the combination of these characterization techniques, we believe that the amorphous surface to be comprised of a mixture of tin- and sodium-containing carbonates and oxides.

Rietveld refinements were initially performed using the stoichiometric ratio of the elements as designed, with good fitting obtained (Table S2 (ESI†)). To investigate the tin content in bulk structure, the occupancy of the B site was allowed to refine, the total occupancy of the B site was fixed at 1, the ratio of Ni, Mn, and Fe was fixed at 1:1:1, and the sodium vacancy linked to the Sn content. The resulting crystal structure parameters are shown in Table S3 (ESI†). In most cases, the Sn content refined to slightly lower than starting precursor ratio, supporting the observation of the Sn/Na/O composite forming on the surface of the particles.

XPS spectra were conducted to investigate the influence of Sn modification on the component and chemical valence. According to the survey spectrum (Figure S9a, ESI†), all of the samples show similar peaks, except for the extra peak of Sn located at 486.5 eV in Sn3- and Sn8%-modified samples (Figure S9a–c, ESI†), which corresponds to the existence of Sn^4+^ (486.7 eV in the standard SnO_2_ and Na_2_SnO_3_).^62^ The high-resolution XPS spectra of all elements are shown in Figure 3. The Ni 2p spectrum (Figure 3a) is dominated in the three cases by an intense spin-orbit doublet characterized by binding energies (BE) of the Ni 2p_3/2_ and Ni 2p_1/2_ core levels of 854.2 and 871.5 eV, respectively. The spectra also show intense shake-up satellites at 860.8, 864.7, and 878.9 eV. Since the main photoemission lines are slightly asymmetric in their high binding energy side as a result of unresolved multiplet splitting, two additional components (labeled MS comp. in the figure) at 857.8 and 875.6 eV were added to the fit to account for this asymmetry. All of these binding energy values are compatible with the presence of Ni^2+^.^63,64^ The Fe 2p spectra (Figure 3b) also show a spin-orbit doublet with BE (Fe 2p_3/2_) =710.4 eV and BE (Fe 2p_1/2_) =723.9 eV and a small shake-up satellite at 718.3 eV. Similarly to the Ni case, we have simulated the asymmetry in the high binding energy of the main component of the doublet induced by unresolved multiplet splitting by adding a peak at 713.8 eV. All of these BE values and spectral features are characteristic of high spin Fe^3+^.^65,66^ In the case of samples Sn3%-NaNFMO and Sn8%-NaNFMO, there appears an additional peak at 715.0 eV, which corresponds to the Sn 2p_3/2_ core level of the tin these samples have been doped with. Finally, the Mn 2p spectra (Figure 3c) show a broad spin-orbit doublet, BE (Mn 2p_3/2_) = 641.8 eV and BE (Mn 2p_1/2_) = 653.4 eV, which is characteristic of the presence of Mn^4+^.^63,65^ The presence of Mn^4+^ is confirmed by the energy separation between the Mn 3s peaks (split as a consequence of multiplet splitting) which is 4.2 eV, see Figure 3d. A remarkable feature of all of these spectra is that no obvious difference was observed with Sn modification, which means TM oxidation states on the surface are essentially identical, indicating likely charge compensation through low-level sodium vacancies on the A site with Sn doping rather than a change in oxidation state of the transition metals on the B site.

The recorded C 1s and O 1s spectra are shown in Figure 3e,f. The C 1s spectra can be fitted to five different carbon contributions located at 282.1, 284.6, 286.2, 288.9, and 290.8 eV, which can be associated with carbides, C–C bonds, C–O–C bonds, carbonates, and O–C=O groups, respectively.^62,67,68^ The O 1s spectra were fitted to four different components located at 528.7, 530.8, 532.8, and 535.3 eV corresponding to metal–oxygen bonds, carbonates/OH groups, organic carbon, and adsorbed water, respectively.^69−71^ In particular, it is very remarkable that the intensity of the carbonate contribution decreases; it amounts to 46% in the pristine NaNFMO and down to 20 and 24% in Sn3%-NaNFMO and Sn8%-NaNFMO, respectively. The decrease in the carbonate contribution is also reflected in the O 1s spectra and Na KLL spectra (Figure S9c, ESI†). The modified surface may explain the lower concentration of sodium carbonates on the surface.

Mössbauer spectroscopy was used to further verify Fe^3+^. The Mössbauer spectra recorded from these samples consisted of a paramagnetic quadrupole doublet (Figure S10a, ESI†). The spectra were best fitted to distribution of quadrupole split absorptions (Figure S10b, ESI†) with values of chemical isomer shift (0.34 mm/s). The distributions show relative maxima at around 0.54 and 0.90 mm/s with an average value of 0.65 mm/s, which are characteristic of Fe^3+^ in octahedral oxygen coordination.^72,73^ This is consistent with the XPS data. The fitting to a distribution model as opposed to discrete one or two doublets indicates that while Fe^3+^ ions adopt essentially identical octahedral sites, the electronic environments of the Fe^3+^ ions experience perturbation from the nearest neighbor or next nearest neighbor ions. The data indicate that the introduction of increasing concentrations of Sn^4+^ enhances the heterogeneity of these minutely different electronic environments, presumably as a result of the higher charge on Sn^4+^.

This detailed compositional analysis indicates that simultaneous bulk and surface modifications occur in these samples. Sn^4+^ substitutes onto the B site lattice, with no change to the oxidation states of the other transition metals, forming sodium-ion vacancies upon the A site. The remaining sodium and tin form a Sn/Na/O rock-salt composite coating upon the particle surface.

### 3.2 Electrochemical Performance

**Figure 4:**
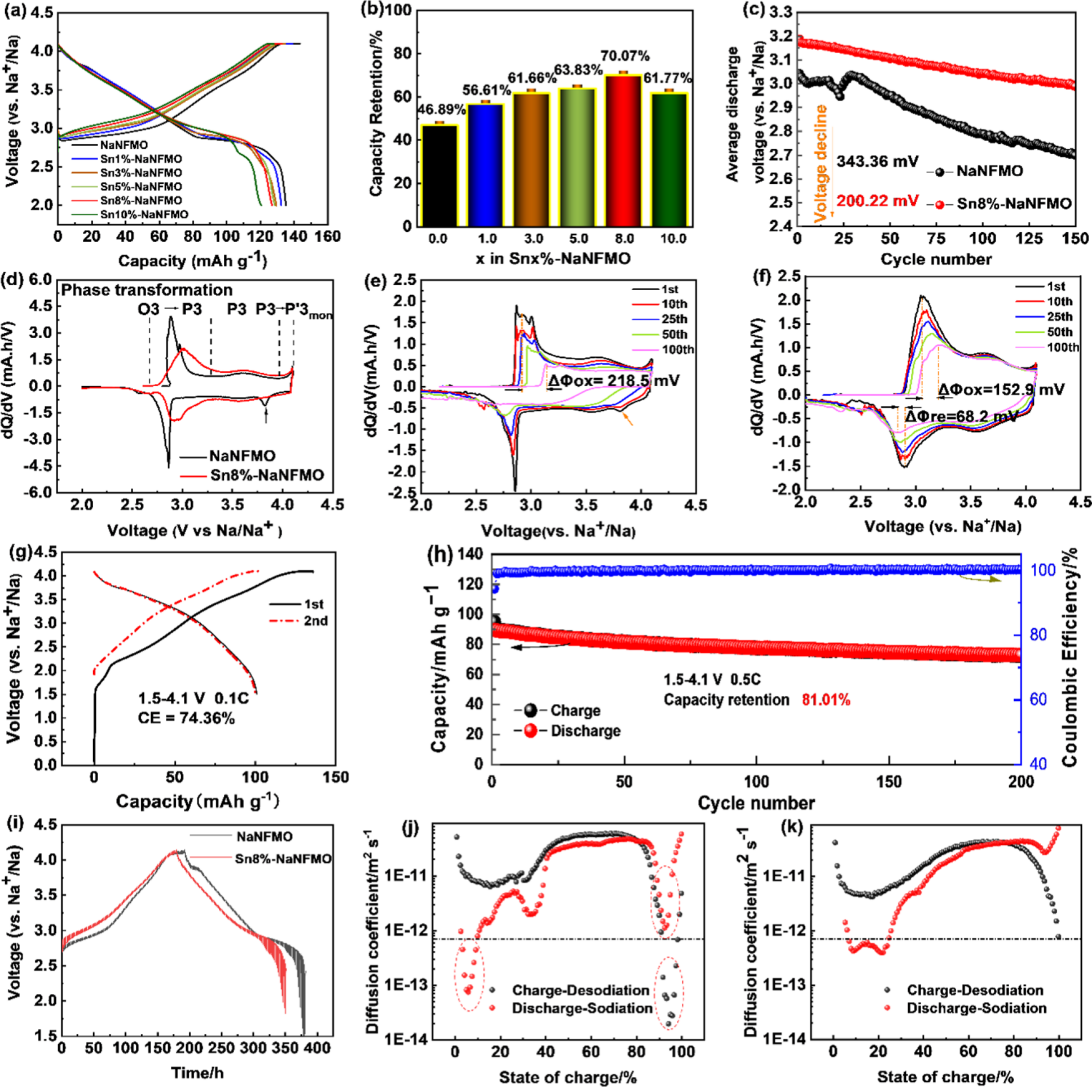
Electrochemical performance of the as-synthesized materials. (a) Initial charge–discharge curves at room temperature; (b) capacity retention of all of the electrodes after 150 cycles at 75 mA/g (0.5C); (c) average discharge voltage of NaNFMO and Sn8%-NaNFMO at 0.5C; (d) comparison of initial differential capacity (d*Q*/d*V*) curves of NaNFMO and Sn8%-NaNFMO at 0.1C; d*Q*/d*V* curves of (e) NaNFMO and (f) Sn8%-NaNFMO cathodes under various cycles at 0.5C; (g) capacity voltage curves of the initial two cycles at 0.1C and (h) cycling performance at 0.5C between 1.5 and 4.1 V in the full cell; and (i) GITT curves of NaNFMO and Sn-NaNFMO cathode material in the first cycle within 2.0–4.1 V; Na diffusivity versus the state of charge and discharge calculated by GITT of (j) NaNFMO and (k) Sn8%-NaNFMO.

The electrochemical properties of the synthesized cathodes were evaluated in the voltage range of 2.0-4.1 V *vs* Na/Na^+^. Figure 4a shows the initial galvanostatic charge/discharge curves of the prepared cells with a current density of 15 mA g^–1^ (0.1C). The initial discharge capacity decreases as the Sn content increases, giving 135.13, 132.46, 129.50, 128.60, 126.91, and 120.32 mAh g^–1^ for NaNFMO, Sn1%-NaNFMO, Sn3%-NaNFMO, Sn5%-NaNFMO, Sn8%-NaNFMO, and Sn10%-NaNFMO, respectively, partially due to the higher molecular weight of Sn compared to Ni, Fe, and Mn, and A site sodium vacancies produced by charge balance of Sn^4+^ on the B site. The Sn-modified electrodes exhibit higher charge/discharge voltage over the potential range than the pristine one and show a smoother voltage profile, indicating greater solid solution phases, rather than phase changes occurring as the sodium intercalates and deintercalates (except the one with 10% Sn), as shown in Figure S11a,b (ESI†). Lower polarization and enhanced rate capability are also observed (Figure S11c,d, ESI†).

To understand the effects of Sn modification on the long-term cycling performance, after three cycles at 15 mA g^–1^, all of the cells were cycled between 2.0 and 4.1 V *vs* Na/Na^+^ at a current density of 75 mA g^–1^ (0.5C) for 150 cycles. As shown in Figure S11e (ESI†) and Figure 4b, although the pristine NaNFMO cathode showed the highest primary capacity (118.95 mAh g^–1^), it shows a rapid decrease with cycling, leading to a capacity retention of only 46.89% after 150 cycles. In contrast, Sn-modified samples show gradually improved capacity retentions, and Sn8%-NaNFMO displays the best cycling performance with a capacity retention of 70.07% for the same cycling period, which was much higher compared to NaNFMO. In addition, the Sn8%-NaNFMO electrode offered the highest and most stable Coulombic efficiency among all during the whole cycling period. These results indicate that appropriate SnO_2_ modification can greatly improve the electrochemical performance of O3 layered oxides. Sn-free NaNFMO and Sn8%-NaNFMO are further compared in detail.

Figure 4c compares the variation of average discharge voltage with cycling between NaNFMO and Sn8%-NaNFMO. The average discharge voltage notably rises from 3.04 to 3.19 V *vs* Na^+^/Na with 8%Sn modification. As previous reports, the rising of the average voltage is caused by the increase of ionicity of the B–O bond in the O3-type ABO_2_, after Sn substitution through reduced d orbital overlap between transition metals and oxygen within the MO_2_ slabs.^46,47,49^ The average voltage upon discharge of NaNFMO dropped significantly from 3.04 to 2.70 V *vs* Na/Na^+^ with a retention rate of 88.70% after 150 cycles at 0.5C. Encouragingly, Sn8%-NaNFMO exhibits excellent voltage retention of 93.72%. The average voltage only dropped by 200 mV from 3.19 to 2.99 V (*vs* Na^+^/Na), compared to 343.35 mV of NaNFMO. This indicates significantly lower polarization increase upon cycling. The capacity *vs* voltage profiles of NaNFMO and Sn8%-NaNFMO at various cycles are shown in Figure S11f,g (ESI†).

To further verify the improved structural stability of the modified electrode, the d*Q*/d*V* studies were performed. Figure 4d shows the differential capacity curves of NaNFMO and Sn8%-NaNFMO cathodes at the first cycle. The sharper peaks observed in NaNFMO (black line) represented the phase changes within the structure,^74^ which is different from the depressed and broader peaks in Sn8%-NaNFMO (blue line), indicating a greater solid solution limit between phase changes. The first peak around 3.0 V (*vs* Na^+^/Na) can be attributed to the phase transition from O3 to P3. The redox potential of Sn8%-NaNFMO cathode increases from 2.9 to 3.0 V (*vs* Na^+^/Na), which means that the phase transition from O3 to P3 moved to higher voltages. Furthermore, the depressed and broader peaks imply that Sn8%-NaNFMO undertook a temperate transformation between phases, and the O3 phase remained stable for the majority of the Na-ion deintercalation process. Upon charging above 4.0 V (*vs* Na^+^/Na), a new oxidation peak and a corresponding reduction peak around 3.8 V (*vs* Na^+^/Na) (marked as an arrow) are observed for the NaNFMO cathode. It is associated with the conversion of the P3 phase into a new phase, monoclinic P′3 as reported,^28,29^ which consists of a distorted lattice in comparison to an ideal hexagonal cell. In contrast, there is no corresponding reduction peak during 3.85–3.75 V (*vs* Na^+^/Na) of Sn8%-NaNFMO when discharged to 2.0 V (*vs* Na^+^/Na). However, if we change the cut-off voltage to 4.2 V (*vs* Na^+^/Na), the reduction peak appears at the same position as NaNFMO (Figure S11h, ESI†), and the cycling stability decreases dramatically (Figure S11i, ESI†). The higher charging voltage (≥4.2 V) triggers the irreversible P3 → P′3 phase transformation, but the Sn-modified Sn8%-NaNFMO increases this irreversible phase transition to 4.1 V compared to 4.0 V (*vs* Na^+^/Na) for Sn-free NaNFMO sample. Upon cycling, the polarization and irreversibility of the NaNFMO electrode are effectively reduced by Sn modification (Figure 4e,f). The redox peaks for Sn8%-NaNFMO remain even after 100 cycles, while all of the oxidation and reduction peaks essentially disappear for NaNFMO. It is noteworthy that the high-voltage O3-P′3 oxidation peak completely disappeared after only 10 cycles, illustrating the irreversibility of the P3–P′3 phase transition.

The practical application of 8%Sn-modified NaNFMO cathode was further verified in a coin full cell with hard carbon (HC) as an anode. A capacity balance of Sn8%-NaNFMO: hard carbon of 1:1.13 was used, based upon reversible capacity observed in the half-cell (Figure S12, ESI†). The cell underwent formation at 0.1C for two cycles and was subsequently cycled at 0.5C charge and discharged in the voltage range of 1.5–4.1 V *vs* hard carbon (Figure 4g,h). Though a relatively high first cycle loss of around 25% was observed, 81% capacity retention was achieved at 0.5C over 200 cycles. This demonstrates the potential for practical commercial applications.

To examine the influence of Sn modification on the kinetics behavior of Na^+^, GITT (galvanostatic intermittent titration technique) was carried out to determine the apparent diffusion capability of Na^+^ (*D*_Na_) in the initial charge/discharge process based on eq 1 introduced by Weppner and Huggins^75^
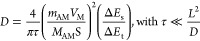
1where *D* is the apparent diffusion coefficient (cm^2^ s^–1^), τ means the applied current time interval, *m*_AM_, *V*_M_, *M*_AM_, and *L* represent the mass of active material, molecular volume, molar weight, and thickness of the electrode, respectively. *S* refers to the electrode effective surface area, which was calculated from the electrode properties and the average particle size. Δ*E*s and Δ*E*_t_ are changes in the steady-state voltage (Δ*E*_s_) and total change of the cell voltage (Δ*E*_t_) during per GITT step (Figure S13, ESI†). More details are listed in Table S4.

The *D*_Na_ calculated from GITT curves (Figure 4i) as a function of the state of charge during both sodiation and desodiation processes are shown in Figure 4j,k. In general, both samples show a similar profile, with a peak of around 70% SoC, and two troughs at the end of charge and discharge, respectively. Both electrodes display almost the same *D*_Na_ value most of the time (10–90% SoC) in the charging and discharging process. However, the Sn-modified sample exhibits an increased *D*_Na_ value at the end of charge and discharge. Specifically, the diffusion coefficient of Na^+^ in the Sn8%-NaNFMO electrode is ∼2.07 × 10^–11^ to 4.41 × 10^–11^ cm^2^ s^–1^ within the voltage window of 2.5–4.0 V and ∼1.88 × 10^–11^ to 7.75 × 10^–13^ cm^2^s^–1^ within the voltage window of 4.0–4.1 V. By contrast, the *D*_Na_ value for the NaNFMO electrode is ∼6.28 × 10^–12^ to 5.99 × 10^–11^ cm^2^ s^–1^ within the voltage window of 2.5–4.0 V (*vs* Na^+^/Na) and ∼1.59 × 10^–11^ to 1.93 × 10^–14^ cm^2^ s^–1^ within the voltage window of 4.0–4.1 V (*vs* Na^+^/Na). The *D*_Na_ value for NaNFMO at the end of charge is only one-tenth of those for Sn8%-NaNFMO, which further indicates that the phase transition in the high-voltage region results in sluggish kinetics for Na ion, and Sn doping suppresses the unfavorable structure change.

**Figure 5:**
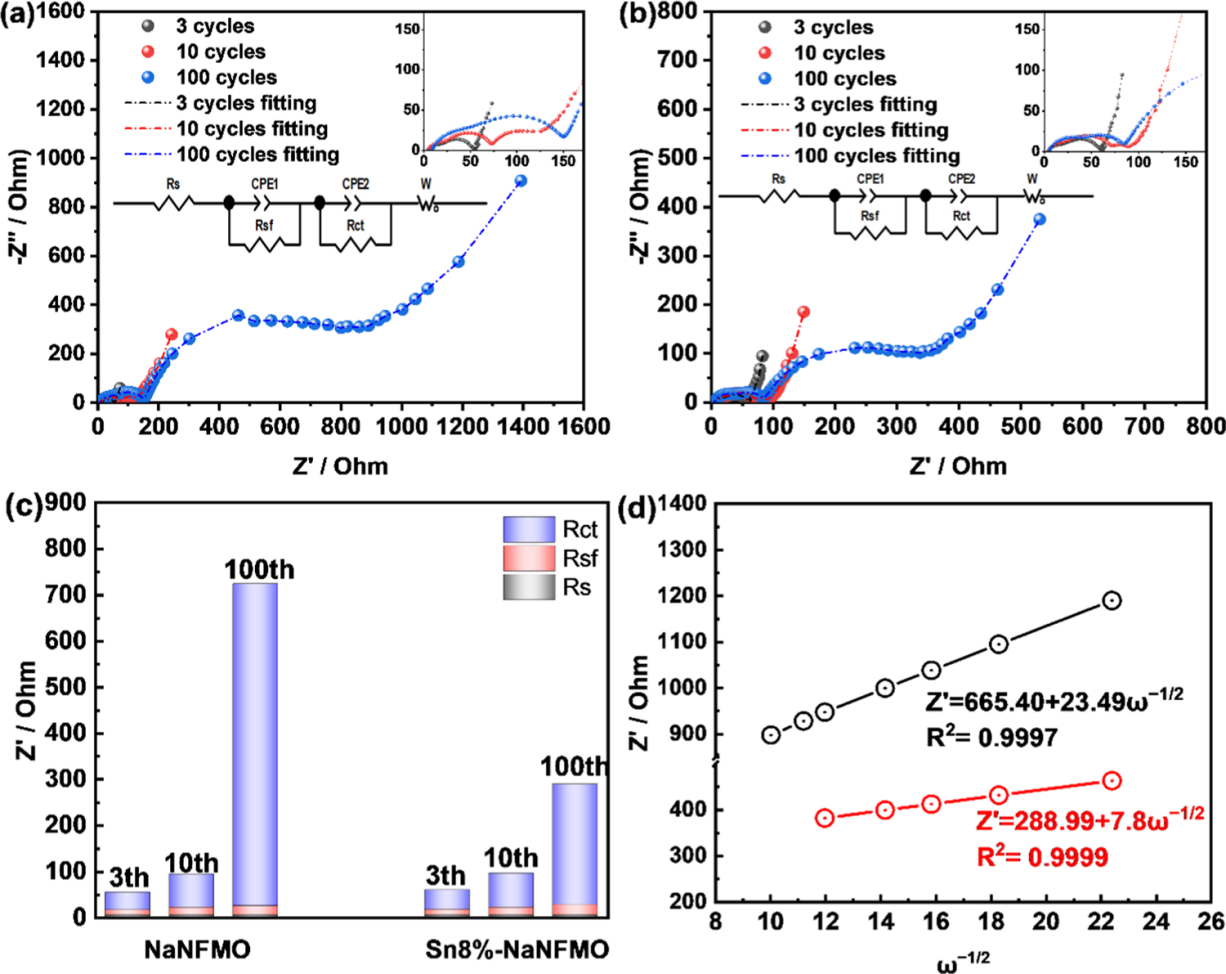
Nyquist plots of (a) NaNFMO and (b) Sn8%-NaNFMO after different cycles. (c) Comparison of fitted impedance for NaNFMO and Sn8%-NaNFMO. (d) Liner fitting of *Z*′ *vs* ω^–0.5^ in the low-frequency region for NaNFMO and Sn8%-NaNFMO samples after 100 cycles.

The interfacial impedance information and the internal Na^+^ diffusion coefficient of the two samples after 100 cycles were analyzed by EIS according to the method reported previously.^76^Figures 5a,b and S14 (ESI†) display the Nyquist and Bode plots of the two samples under a fully charged state with different cycles at 0.5C, and the equivalent circuits fitted by *Z* View 2.0 software are inserted. The small interrupt at the beginning corresponds to the solution resistance *R*e. The two semicircles in the high and middle frequency can be attributed to the electrified interface resistance (*R*_sf_) and the charge-transfer resistance (*R*_ct_) between the electrolyte and electrode, respectively. The approximate straight line in the low frequency reflects the Na^+^ diffusion impedance within the cathode bulk crystal (*W*s).^77^ The fitted parameters are shown in Figure 5c. The Sn-modified sample displays a much lower increase in *R*_ct_ impedance than the pristine sample after 100 cycles, which indicates that the interface side reaction is significantly suppressed due to the enhanced crystal structure as well as the modified surface. The calculated *D*_Na_ is 1.59 × 10^–12^ cm^2^ s^–1^ for Sn8%-NaNFMO after 100 cycles, which is much higher than 2.17 × 10^–13^ cm^2^ s^–1^ for NaNFMO. The smaller impedance and higher *D*_Na_ signify the structure of Sn8%-NaNFMO remain well ordered after an extensive cycle. Combining the results of GITT and EIS, we can conclude that the Sn modification has a positive effect both on the kinetics behavior of Na^+^ and the structural stability of the material after cycling.

### 3.3 Mechanism Investigation

**Figure 6:**
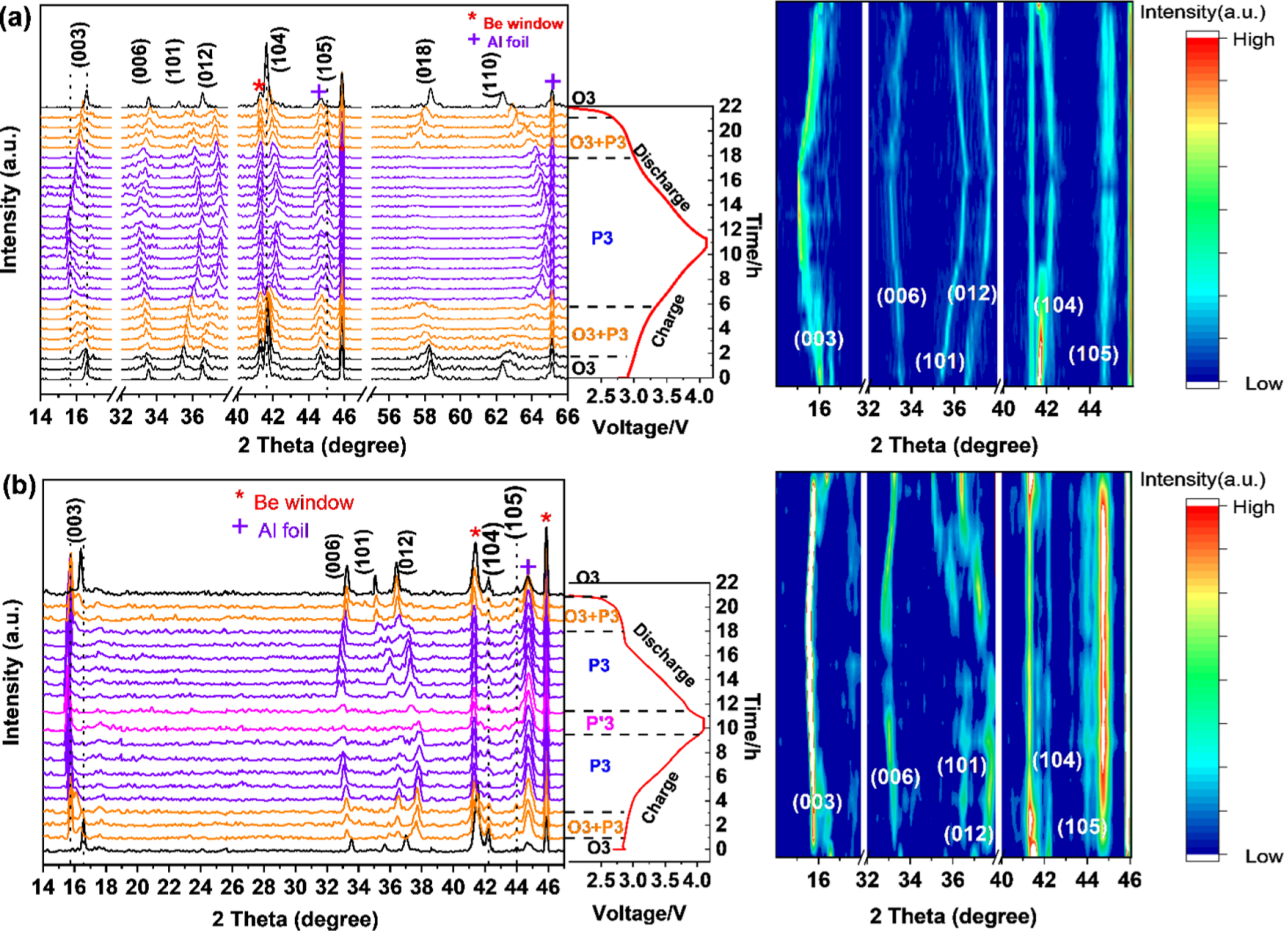
Operando XRD patterns and corresponding 2D contour maps showing the evolution of the main characteristic peaks for (a) Sn8%-NaNFMO and (b) NaNFMO cathodes during the first charge/discharge process between 2.0 and 4.1 V at 0.1C.

To gain insight into the effects of Sn modification on structural evolution upon Na^+^ extraction/insertion, operando XRD analysis was performed for NaNFMO and Sn8%-NaNFMO electrodes during the first cycle in the voltage range of 2.0–4.1 V (*vs* Na^+^/Na) at 0.1C. Figure 6 shows the corresponding XRD patterns collected at various charge and discharge depths in Na half-cells. Generally, this peak variation is in good agreement with the previous report, demonstrating an O3_hex_ → P3_hex_ → (P′3_mon_) → P3_hex_ → O3_hex_ sequence during cycling.^28,55^ Upon initial charging, (003), (006), and (018) peaks shift to lower angles, while (101), (012), (104), and (110) peaks shift to higher angles for both samples, indicating the increase of *c*-axis and decrease of *a*-axis in the O3 phase. When charging to 3.2 V (*vs* Na^+^/Na), the NaNFMO sample experiences the first phase transition from hexagonal O3 to hexagonal P3 phase, which was evident from the fact that the (003) peak shift from 16.5 to 16.0° and a significant decrease in peak intensity of (104), as well as the appearance of (105) peak. In contrast, the O3 phase of Sn8%-NaNFMO remains until 3.3 V, indicating that the phase transitions from hexagonal O3 to hexagonal P3 were delayed by the Sn modification, which was consistent with the dQ/dV curve. Significant differences between the two cathodes were observed when it came to the end of the charge state. With further charging to 4.0 V (*vs* Na^+^/Na), the peak profile of the P3 phase becomes asymmetrical for NaNFMO, accompanied by the disappearance of (006)_Hex.P3_ and (105)_Hex.P3_ peaks. Such peak changing indicates structural deterioration, which can be assigned to the monoclinic P′3 phase with a distorted lattice observed in previous literature.^38^ In contrast, (006)_Hex.P3_ and (105)_Hex.P3_ peaks in the P3 phase still remain for Sn8%-NaNFMO until further charging to 4.2 V (*vs* Na^+^/Na) (Figure S15a, ESI†), indicating that the unfavorable high-voltage phase transformation was suppressed. Upon discharge, all of the peaks can shift back to the initial positions, indicating a reversible process in the first cycle. The evolution of lattice parameters was derived by Rietveld refinement and reported in Figure S15b (ESI†).

Postmortem analyses were conducted to examine the improved structural stability of the Sn8%-NaNFMO cathode. Figure S16a (ESI†) shows the XRD patterns of the pristine and 8%Sn-modified cathode after 150 cycles. The data for NaNFMO shows a significant reduction in peak intensity along with evidence for some peak splitting (particularly for the (003) reflection), indicative of significant structural changes. This is further shown in the Raman data with the emergence of the characteristic peak of spinel-like structure around 660 cm^–1^,^78^ as shown in Figure S16b (ESI†). In contrast, the positions and intensities of the XRD and Raman peaks of Sn8%-NaNFMO were well maintained. The morphology of the cathodes after 150 cycles under 0.5C were observed by SEM. As shown in Figure S17 (ESI†), although no immediate difference was observed, at a closer inspection of the particles from the nondoped sample (Figure S17b,e) surface degradation is observed. Particularly in Figure S17e, the porous particle shows an interface grown into the middle of the sample, while Figure S17b shows a “glue”-type surface coating. In the Sn8% cathode (Figure S17f), there are still open central particle pores that are not filled by this cathode interface growth, and Figure S17d shows less apparent surface degradation on the particles. These results indicate the enhanced structural and surface stability during long-term cycling on Sn incorporation.

## 4 Conclusions

The successfully synthesized novel, low-cost Sn-modified O3-type cathode material (Sn8%-NaNFMO) has been shown to exhibit both good structure stability and electrochemical properties via an industrially feasible coprecipitation method. The influence of Sn modification of Na[Ni_1/3_Fe_1/3_Mn_1/3_]O_2_ on the crystal structure and electrochemical properties has been investigated for the first time, and the mechanism was discussed preliminarily. XRD test and refinement indicated that the Sn-modified sample kept the same O3-type crystal structure of the pristine Na[Ni_1/3_Fe_1/3_Mn_1/3_]O_2_ with enlarged *d*-spacing of the Na^+^ layer. Electrochemical analyses demonstrated that Sn modification was effective in suppressing the phase transformation and thus improving the structure stability significantly, leading to a highly improved Na storage behavior in terms of average voltage, rate capability, and cycling stability. As a result, the 8%Sn-modified NaNFMO cathode exhibited its optimized cycling performance by an almost doubled capacity retention increase after 150 cycles in the wide voltage range of 2.0–4.1 V (*vs* Na^+^/Na). This work demonstrates that controlled Sn modification is a practical and efficient strategy for stabilizing O3-type layered structures for sodium-ion batteries.

## Supporting Information Available

Crystallographic data (CIF)
Schematic illustration of synthesis processes and the positive effects of Sn modification, XRD patterns and Rietveld refinement results, variation of lattice parameters, Raman spectra, SEM images and EDS mapping for Sn8%-NaNFMO, SAED patterns of the pristine material, XPS and Mössbauer spectra, additional electrochemical test results (rate capability, discharge capacity retention of all samples vs cycle number, d*Q* d*V* curves and cycling performance in the voltage range of 2.0–4.2 V, electrochemical performance of hard carbon used in full cell), bode impedance plots, XRD patterns of Sn8%-NaNFMO between 2.0 and 4.2 V, XRD patterns and Raman spectra after 100 cycles, SEM images after 150 cycles chemical composition determined by ICP-OES, and cell parameters used for GITT calculation (PDF)

## Supplementary Material

cm2c00522_si_001.cif
