# Supplementary material for: High-Voltage Stabilization of O3-Type Layered Oxide for Sodium-Ion Batteries by Simultaneous Tin Dual Modification
Source: Chem Mater. 2022 Apr 29;34(9):4153–65. doi: 10.1021/acs.chemmater.2c00522 (PMC9097156; doi:10.1021/acs.chemmater.2c00522)
Supplement: Supplementary file 2 — cm2c00522_si_002.pdf [file cm2c00522_si_002.pdf]

Supporting Information

High-Voltage Stabilization of O3-Type Layered Oxide for Sodium-Ion Batteries by Simultaneous Tin Dual Modification

Tengfei Song [a‡], Lin Chen [a], Dominika Gastol [a, d], Bo Dong [b, d], José F. Marco [c], Frank Berry [b], Peter Slater [b, d], Daniel Reed [a ,d] and Emma Kendrick [a, d,]*

[a] School of Metallurgy and Materials, University of Birmingham, Edgbaston, Birmingham, B15 2TT, UK.

[b] School of Chemistry, University of Birmingham, Edgbaston, Birmingham, B15 2TT, UK.

[c] Instituto de Química Física "Rocasolano", CSIC, Serrano 119, Madrid 28006, Spain.

[d] The Faraday Institution, Harwell Science and Innovation Campus, Didcot, OX11 0RA, UK

[*]Corresponding author: *E.Kendrick@bham.ac.uk*

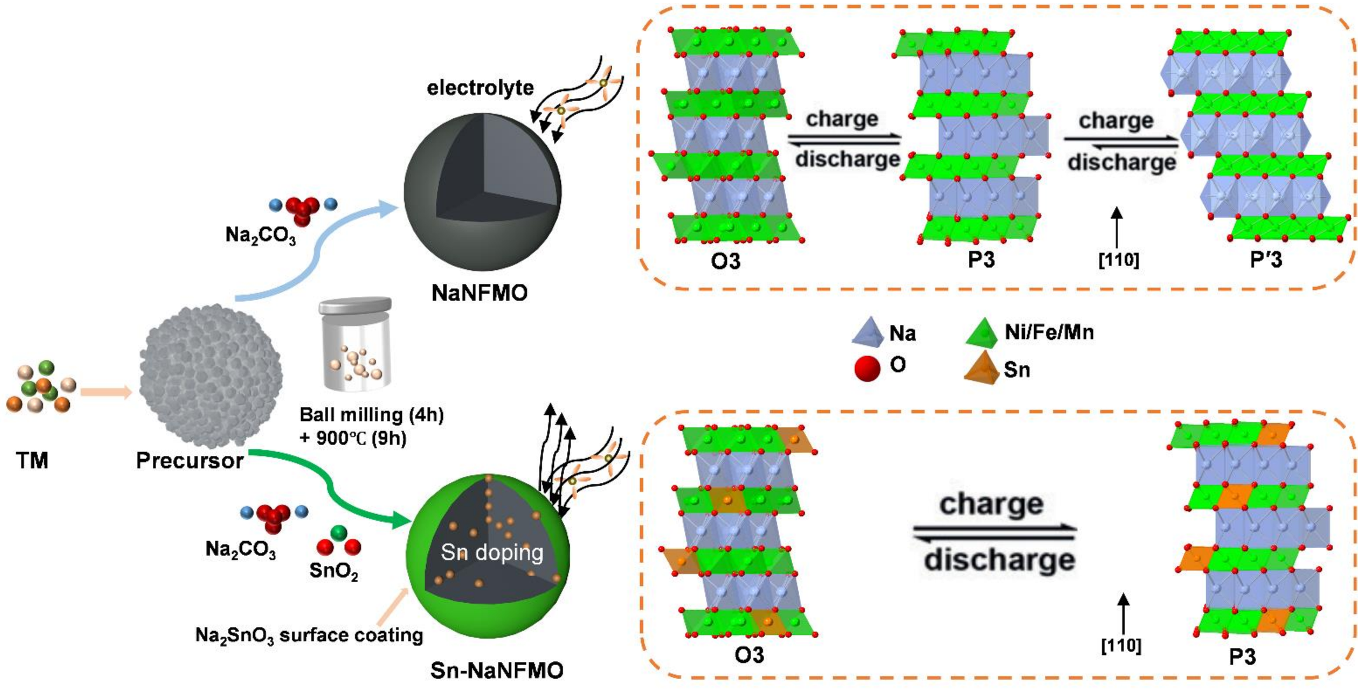

Figure S1. Schematic illustration of synthesis processes and the positive effects of Sn simultaneously dual modification on the O3 NaNFMO cathode's stability during cycling under high voltage.

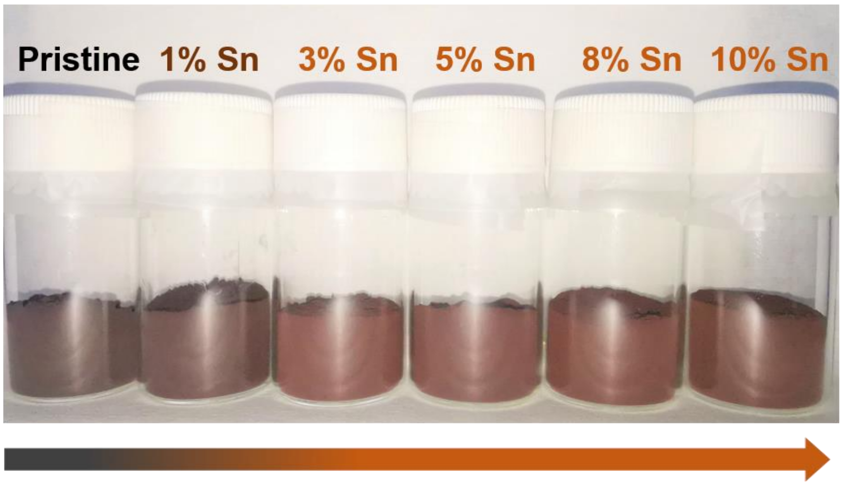

Figure S2. The colour of the different Sn stoichiometry doping layered oxides.

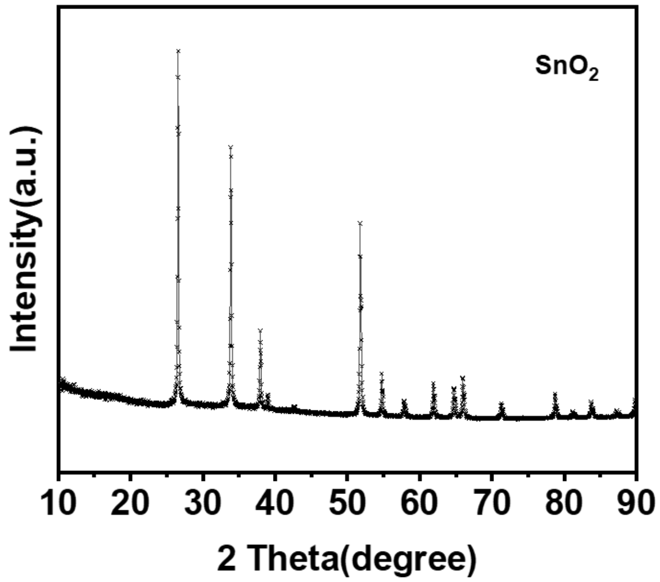

Figure S3. XRD pattern of the pure SnO_2 powder material.

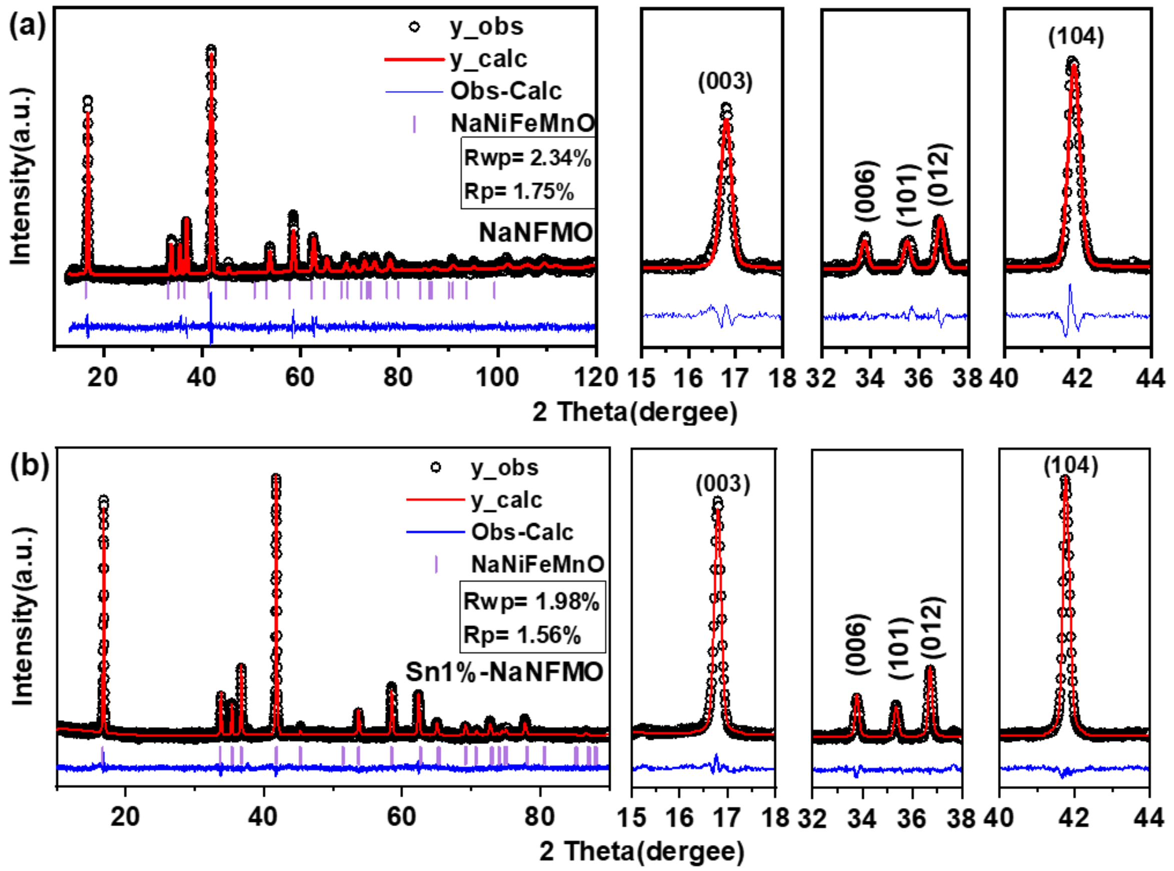

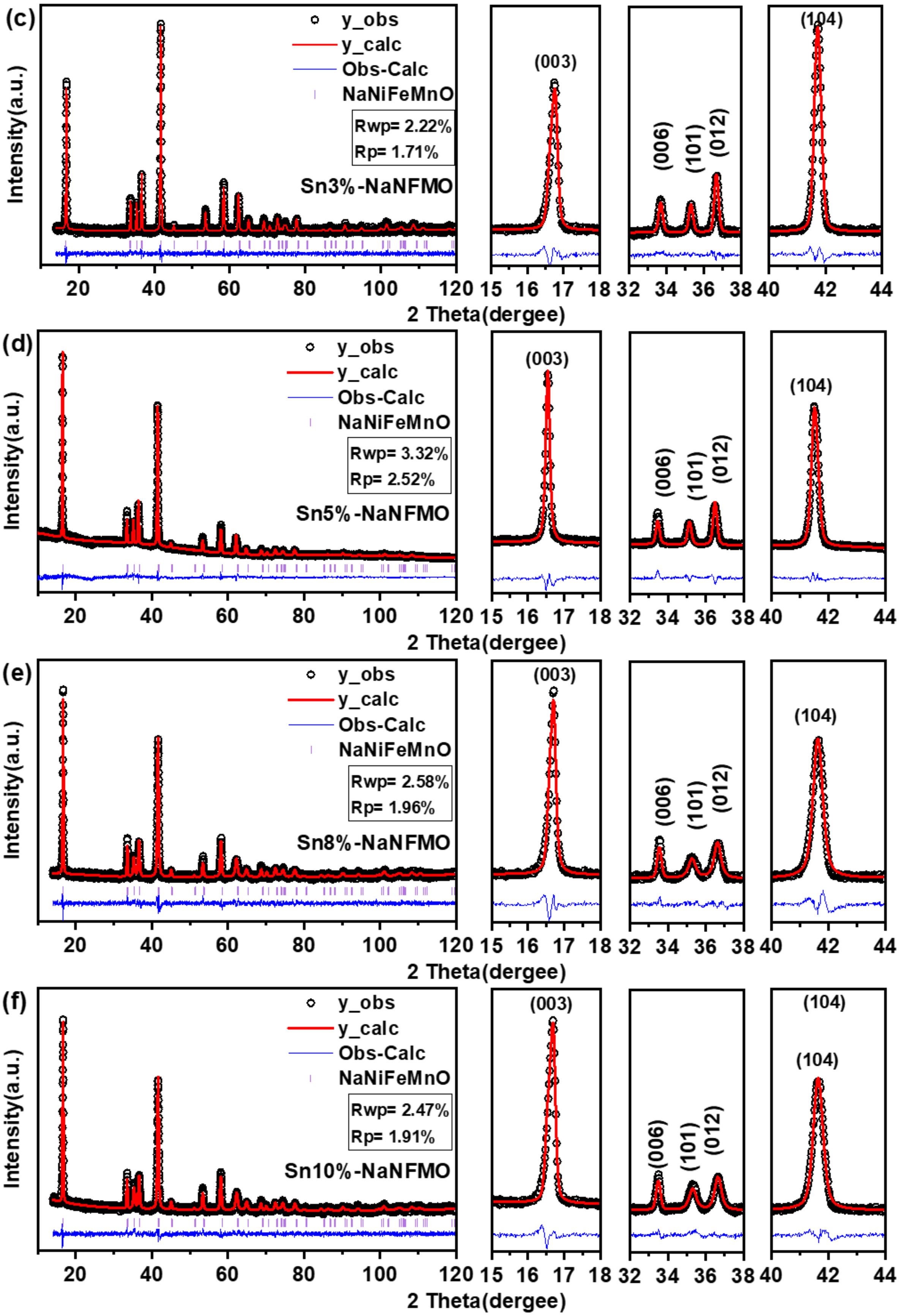

Figure S4. Detailed Rietveld refinements of XRD pattern of the as-prepared cathode powdered materials.

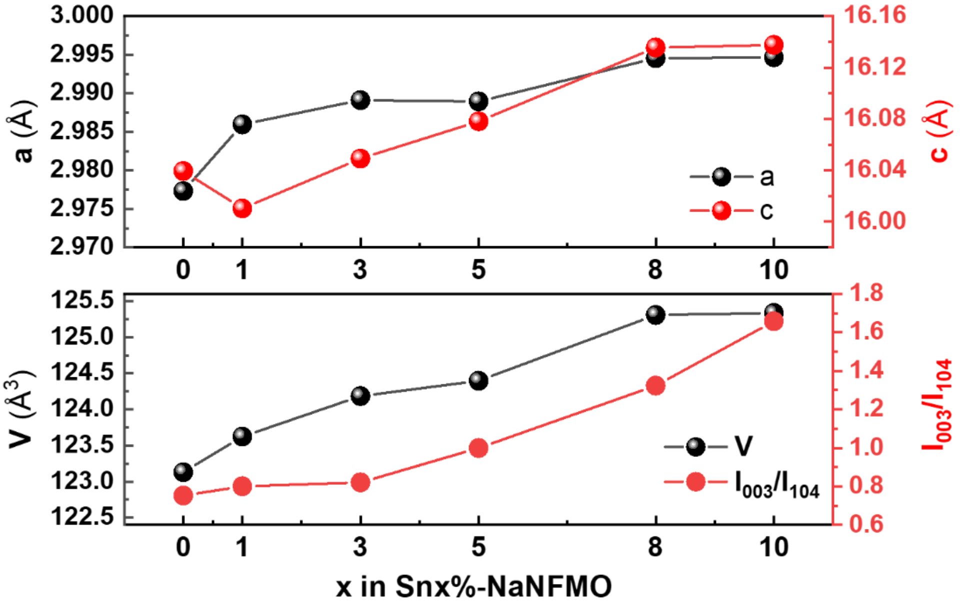

Figure S5. Variation of lattice parameters and the I003/I104 value as a function of SnO_2 content.

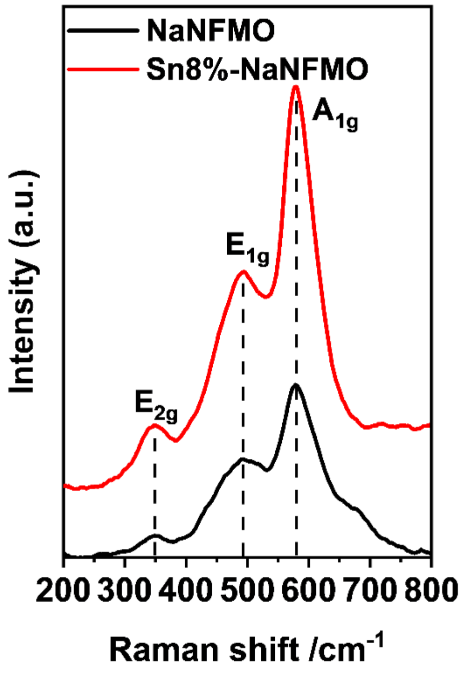

Figure S6. Raman spectra of NaNFMO and Sn8%-NaNFMO.

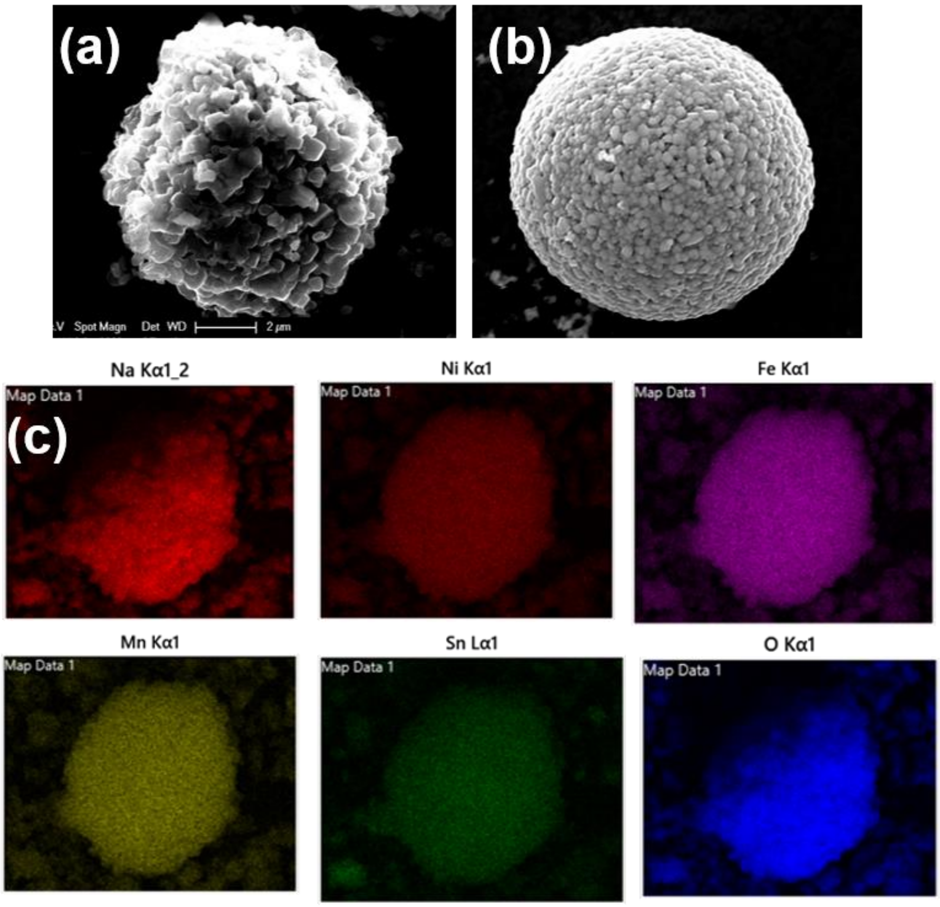

Figure S7. SEM images of (a) NaNFMO and (b) Sn8%-NaNFMO. (c) Corresponding EDS elemental mapping for Sn8%-NaNFMO.

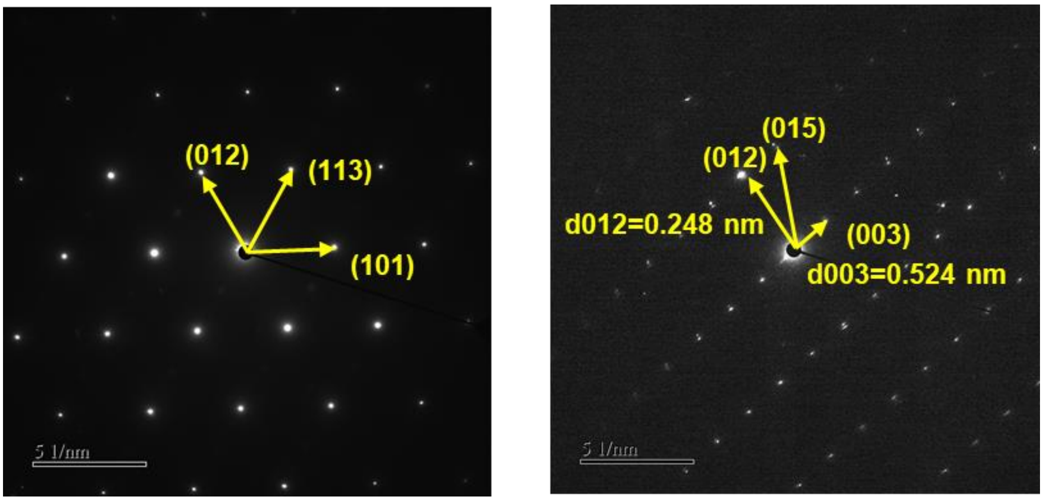

Figure S8. Selected area electron diffraction (SAED) patterns of the obtained NaNFMO material.

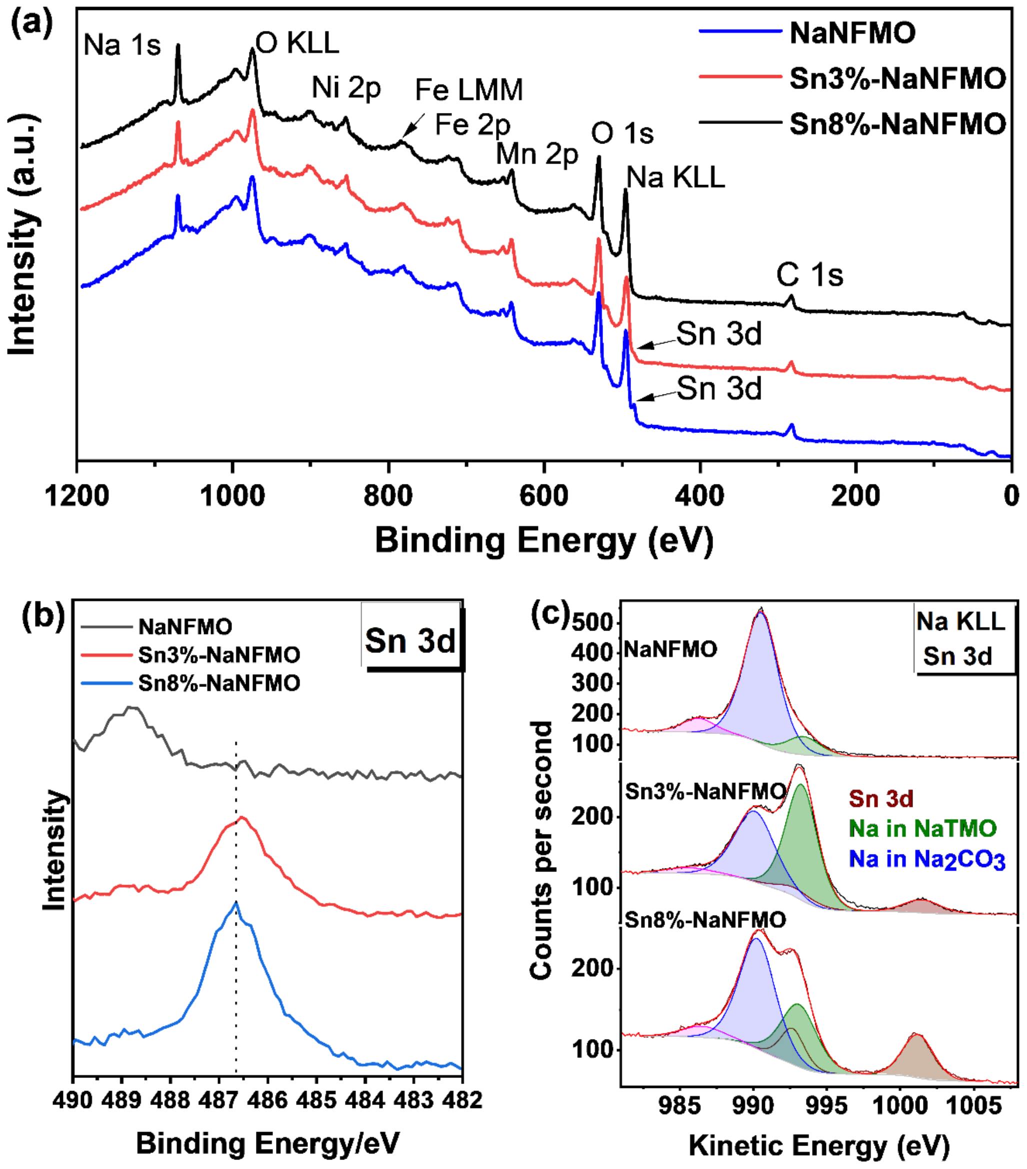

Figure S9. XPS data for the surface of NaNFMO with 0, 3 and 8% Sn additions. (a) Survey XPS spectra (b) Sn 3d5/2 spectra and (c) Na KLL and Sn 3d XPS spectra recorded from pristine NaNFMO, Sn3%-NaNFMO, and Sn8%-NaNFMO.

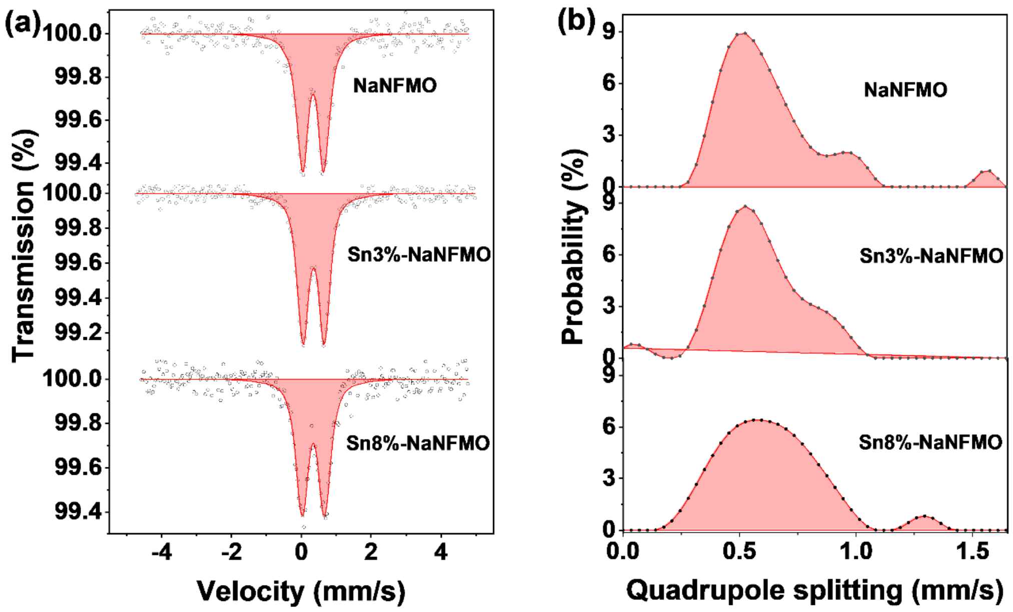

Figure S10. (a) Room temperature ^{57}Fe Mössbauer spectra recorded from pristine NaNFMO, Sn3%-NaNFMO, and Sn8%-NaNFMO. (b) Quadrupole splitting distributions obtained from the fit of the spectra presented on (a).

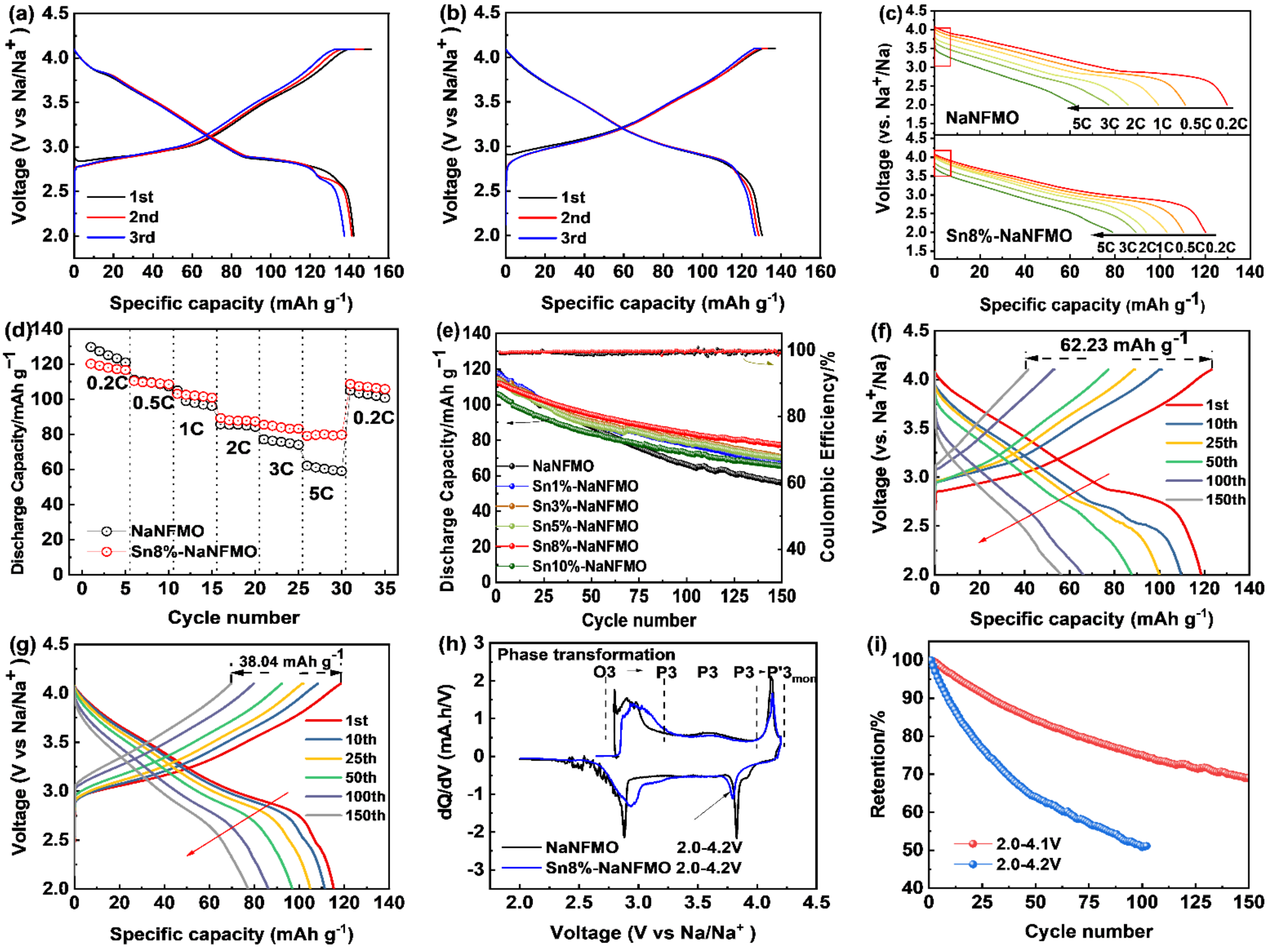

Figure S11. The initial three charge-discharge curves of (a) NaNFMO and (b) Sn8%-NaNFMO. (c) Discharge curves of different current densities for NaNFMO and Sn-NaNFMO. (d) Rate capability at various current densities from 0.2 to 5 C. (e) The cycling performance of all the electrodes at 75 mA/g (0.5C). Charge/discharge curves of different cycles for (f) NaNFMO and (g) Sn8%-NaNFMO. (h) The differential voltage (dQ/dV) curves of NaNFMO and Sn8%-NaNFMO at 2.0-4.2 V. (i) Comparisons of the cycling performance of Sn8%-NaNFMO at different cut-off voltage.

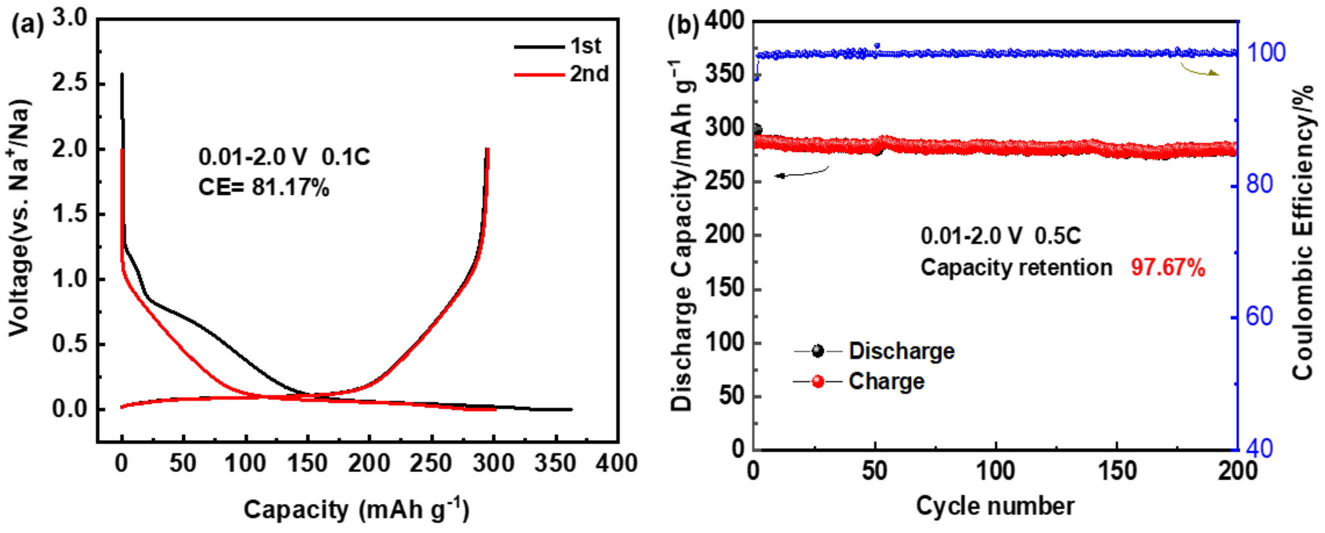

Figure S12. (a) The first initial two voltage curves of the as-prepared hard carbon anode vs. Na^+/Na, showing a reversible capacity of ~300 mAh g^{-1} at a current rate of 0.1C in the voltage range of 0.001-2.0 V and a first cycle loss of 19%. (b) Cycling performance of the commercial hard carbon at 0.5C within 0.01-2.0 V.

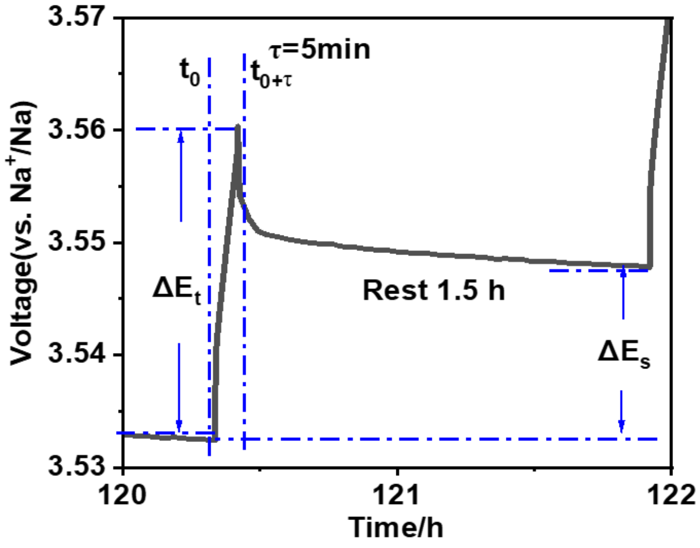

Figure S13. The schematic diagram of a single current pulse for GITT.

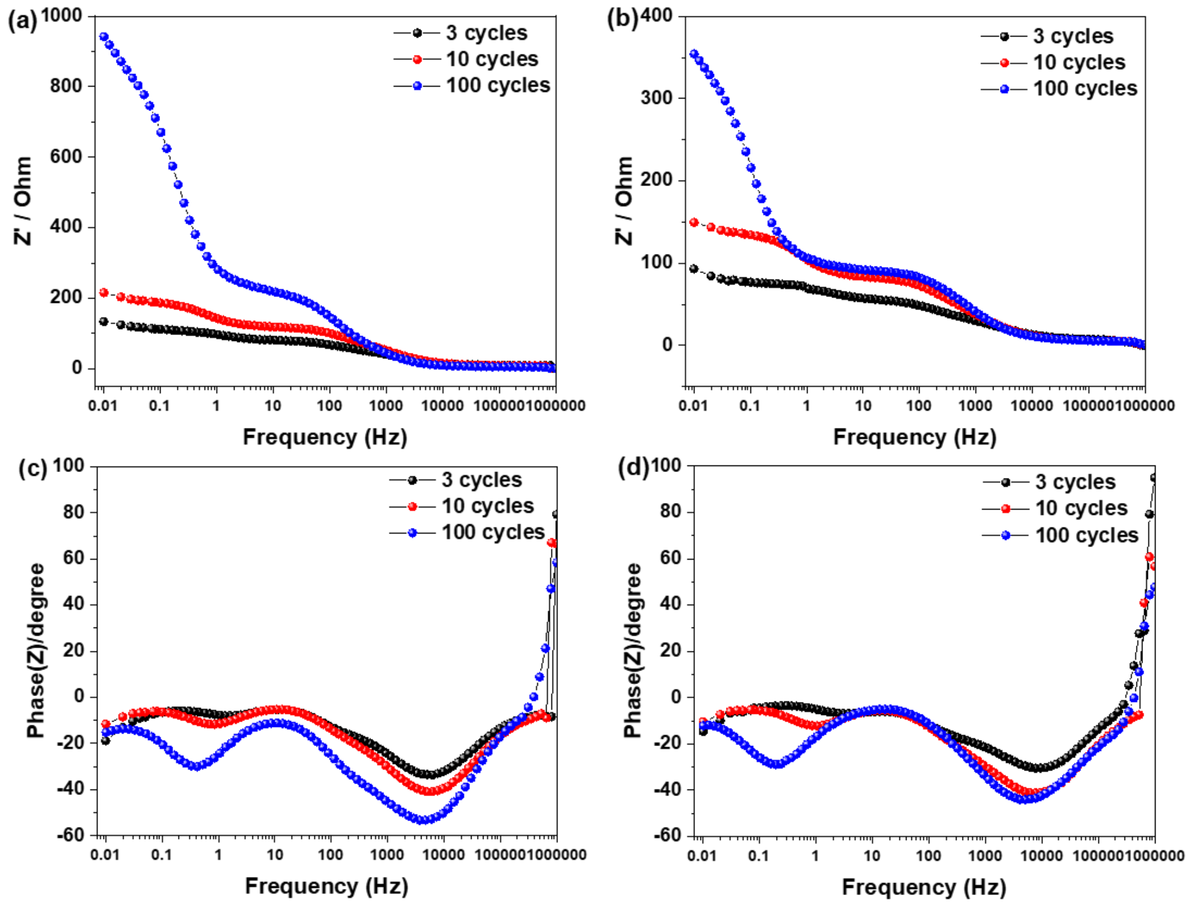

Figure S14. Bode impedance plots of (a) NaNFMO and (b) Sn8%-NaNFMO, and Bode angle plots of (c) NaNFMO and (d) Sn8%-NaNFMO after different cycles.

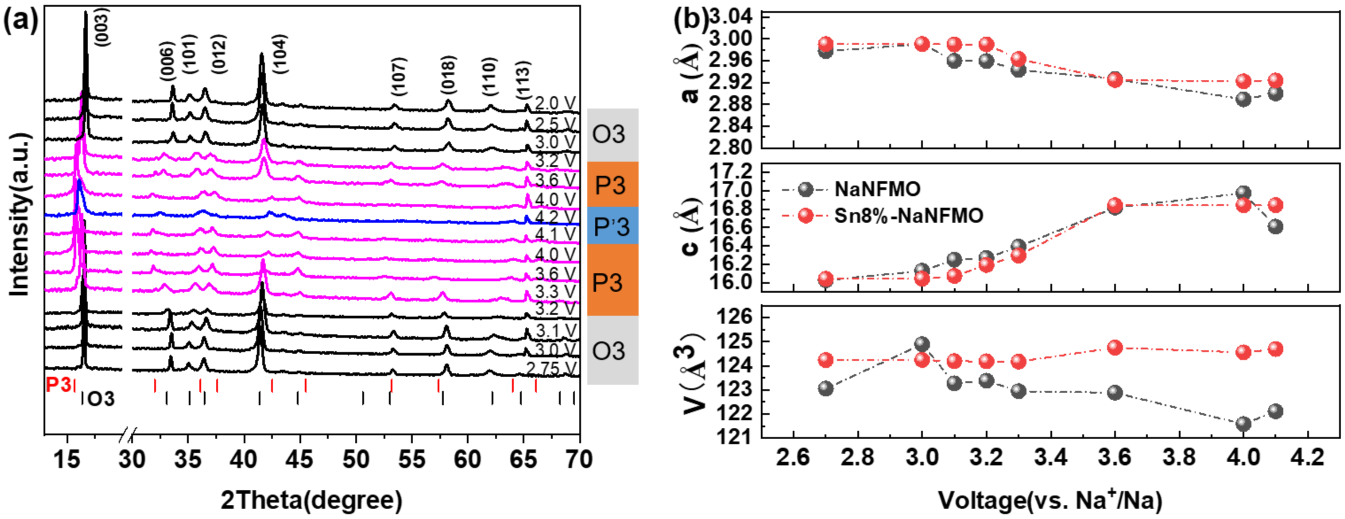

Figure S15. (a) EX-situ XRD patterns during the first charge/discharge of Sn8%-NaNFMO electrodes between 2.0 V and 4.2 V at 0.1 C. (b) Lattice parameters a, c and V evolution during charge process.

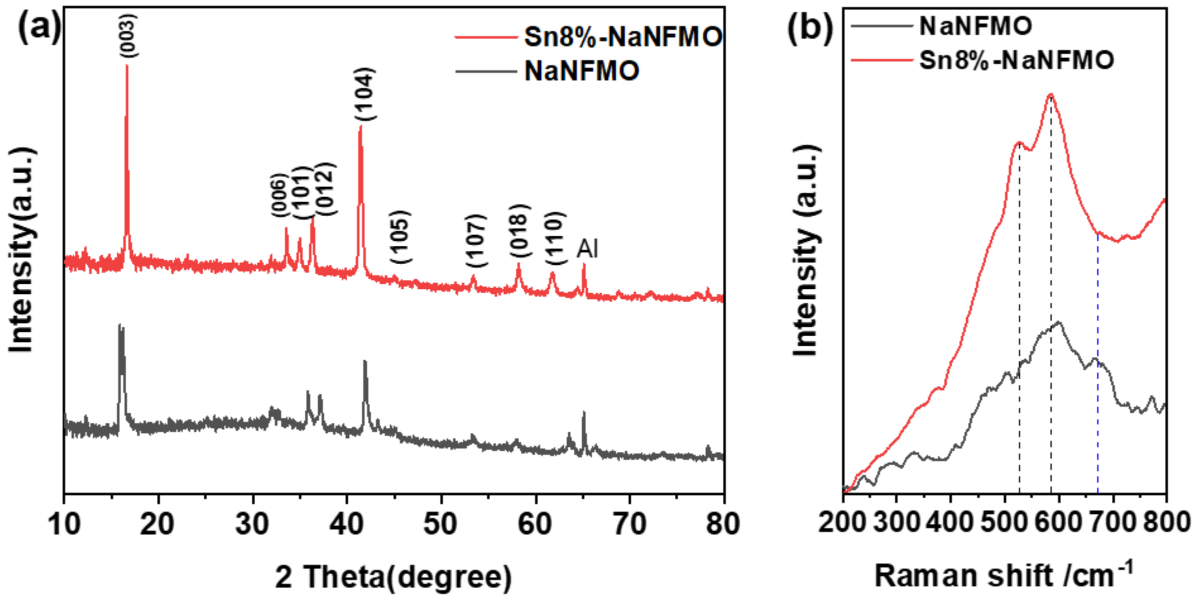

Figure S16. (a) XRD patterns and (b) Raman spectra of NaNFMO and Sn8%-NaNFMO after 100cycles.

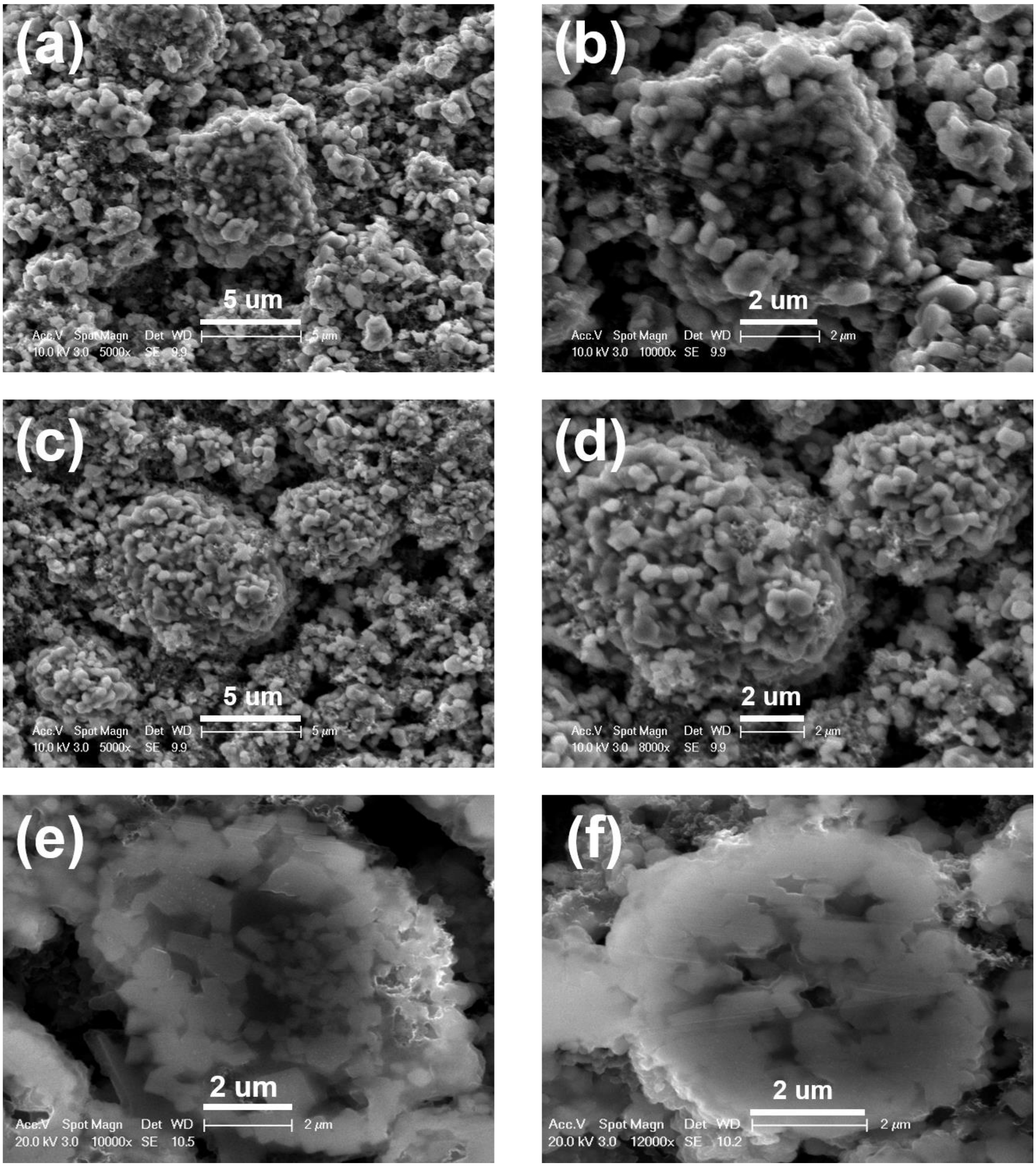

Figure S17. SEM images of NaNFMO (a, b) and Sn8%-NaNFMO (c, d) after 150 cycles. Cross-section images of NaNFMO (e) and Sn8%-NaNFMO (f) after 150 cycles.

Table S1. The chemical compositions of the as-prepared samples measured by inductively coupled plasma mass spectroscopy (ICP-OES).

Compound	Input composition					Final composition				
	Na	Ni	Fe	Mn	Sn	Na	Ni	Fe	Mn	Sn
**$NaMn_{1/3}Fe_{1/3}Ni_{1/3}O_2$**	1.050	0.333	0.333	0.333	0.000	0.998	0.337	0.339	0.324	0.000
**Sn1%-$NaMn_{1/3}Fe_{1/3}Ni_{1/3}O_2$**	1.040	0.330	0.330	0.330	0.001	0.996	0.334	0.337	0.323	0.006
**Sn3%-$NaMn_{1/3}Fe_{1/3}Ni_{1/3}O_2$**	1.019	0.323	0.323	0.323	0.029	0.977	0.324	0.328	0.322	0.026
**Sn5%-$NaMn_{1/3}Fe_{1/3}Ni_{1/3}O_2$**	1.000	0.317	0.317	0.317	0.048	0.929	0.319	0.318	0.311	0.053
**Sn8%-$NaMn_{1/3}Fe_{1/3}Ni_{1/3}O_2$**	0.972	0.308	0.308	0.308	0.074	0.919	0.312	0.313	0.303	0.072
**Sn10%-$NaMn_{1/3}Fe_{1/3}Ni_{1/3}O_2$**	0.955	0.303	0.303	0.303	0.091	1.061	0.309	0.304	0.303	0.084

Table S2. Summary of the refined parameters and the R factors.

	NaNFM	Sn1%-NaNFMO	Sn3%-NaNFMO	Sn5%-NaNFMO	Sn8%-NaNFMO	Sn10%-NaNFMO
**Sn Occupancy**	0.0000	0.0099	0.0291	0.0476	0.0741	0.0909
**TM Occupancy**	0.3333	0.3300	0.3236	0.3175	0.3086	0.3030
**Na Occupancy**	1.0000	0.9901	0.9709	0.9524	0.9259	0.9091
**Rwp/%**	2.34	1.98	2.22	3.32	2.58	2.47
**Rp/%**	1.75	1.56	1.71	2.52	1.96	1.91
**Chi^2**	1.7290	1.59	1.416	0.6448	1.71	1.442
**a(Å)**	2.97732(22)	2.98597(9)	2.98897(8)	2.98895(8)	2.99471(10)	2.99267(10)
**c(Å)**	16.0395(17)	16.0103(7)	16.0480(8)	16.0782(8)	16.1379(12)	16.1681(12)
**c/a**	5.3872	5.3619	5.3692	5.3792	5.3883	5.3888
**V($Å^3$)**	123.132(25)	123.624(10)	124.163(10)	124.395(11)	125.339(14)	125.403(13)
**z for 6c site**	0.23396(23)	0.23009(16)	0.23404(17)	0.23488(17)	0.23780(22)	0.23626(22)
**Na layer(Å)**	3.1878	3.3059	3.1869	3.1659	3.0834	3.1390
**TMO_2(Å)**	2.1587	2.0309	2.1624	2.1935	2.2959	2.2504
**Na-O(Å)**	2.3442(25)	2.3884(18)	2.3488(19)	2.3417(18)	2.3165(23)	2.3343 (24)
**TM-O(Å)**	2.0298(20)	2.0007(13)	2.0364(14)	2.0444(15)	2.0754(19)	2.0619(19)

Table S3. Summary of the further refined parameters and the R factors considering the sodium vacancy.

	NaNFM	Sn1%-NaNFMO	Sn3%-NaNFMO	Sn5%-NaNFMO	Sn8%-NaNFMO	Sn10%-NaNFMO
**Sn Occupancy**	0.0000	0.0759	0.0178	0.0156	0.0331	0.0908
**TM Occupancy**	0.3333	0.3080	0.3277	0.3281	0.3223	0.3089
**Na Occupancy**	1.0000	0.9241	0.9831	0.9844	0.9669	0.9092
**Rwp/%**	2.46	1.93	2.15	3.30	2.57	2.47
**Rp/%**	1.85	1.53	1.67	2.50	1.96	1.91
**Chi^2**	1.9110	1.506	1.321	0.6366	1.692	1.442
**a(Å)**	2.9775(2)	2.98594(9)	2.98897(8)	2.98899(8)	2.99473(10)	2.99267(10)
**c(Å)**	16.040(2)	16.0102(7)	16.0480(8)	16.0783(9)	16.1377(12)	16.1681(12)
**c/a**	5.3872	5.3619	5.3691	5.3792	5.3887	5.4026
**V($Å^3$)**	123.152(3)	123.620(10)	124.163(10)	124.400(10)	125.339(14)	125.403(13)
**z for 6c site**	0.23396(23)	0.23145(18)	0.23372(20)	0.23405(19)	0.23658(27)	0.23625(24)
**Na layer(Å)**	3.1876	3.2630	3.1975	3.1929	3.1217	3.1387
**TMO_2(Å)**	2.1589	2.0738	2.1519	2.1665	2.2575	2.2506
**Na-O(Å)**	2.3281(29)	2.3734(20)	2.3524(21)	2.3509(21)	2.3293(29)	2.3342(26)
**TM-O(Å)**	2.0428(23)	2.0118(15)	2.0336(17)	2.0375(16)	2.0648(24)	2.0620(21)

Table S4. Summary of cell parameters used for GITT calculation

			NaNFM	Sn8%-NaNFMO
τ	the applied current time interval	min	90	90
M_{AM}	atomic weight of active material	g mol^{-1}	111.48	116.46
V_M	the molar volume of active material	cm^3 mol^{-1}	23.32	24.36
m_{AM}	mass of active material	mg	6.78	6.86
L	the thickness of the electrode	um	29	30
S	the electrode surface area	cm^2	16.34	16.56
